# Supplementary material for: Synthesis and Evaluation of the Antitumor Activity of Novel 1-(4-Substituted phenyl)-2-ethyl Imidazole Apoptosis Inducers In Vitro
Source: Molecules. 2020 Sep 18;25(18):4293. doi: 10.3390/molecules25184293 (PMC7570620; doi:10.3390/molecules25184293)

Synthesis and evaluation of the *in vitro* antitumor activity of the novel and potent apoptosis inducers, 1-(4-substituted phenyl)-2-ethyl imidazoles

Zhen-Wang Li, Chun-Yan Zhong, Xiao-Ran Wang, Shi-Nian Li, Chun-Yuan Pan, Xin Wang, and Xian-Yu Sun

^{1}H-NMR spectrum of Compound **3**

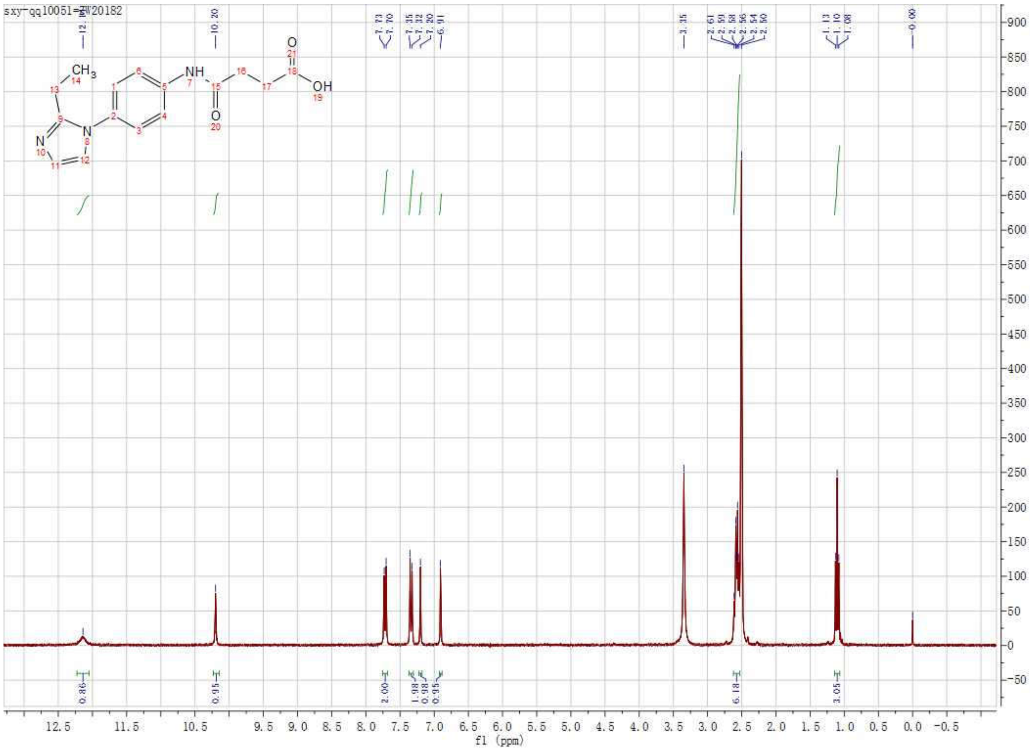

^{1}H-NMR spectrum of Compound **4a**

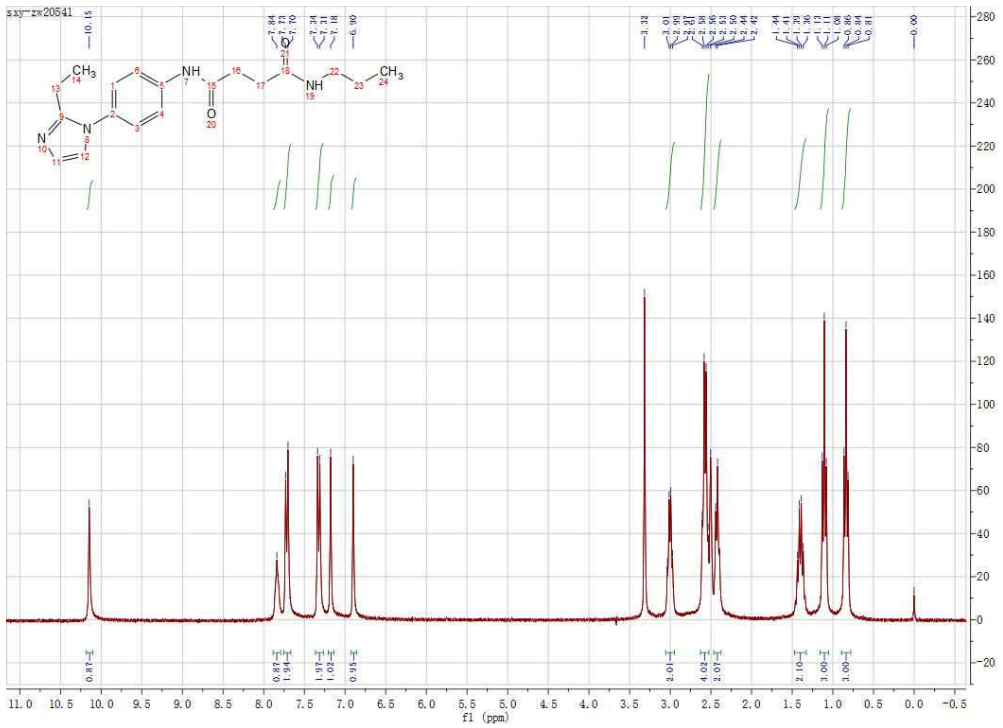

^{13}C-NMR spectrum of Compound **4a**

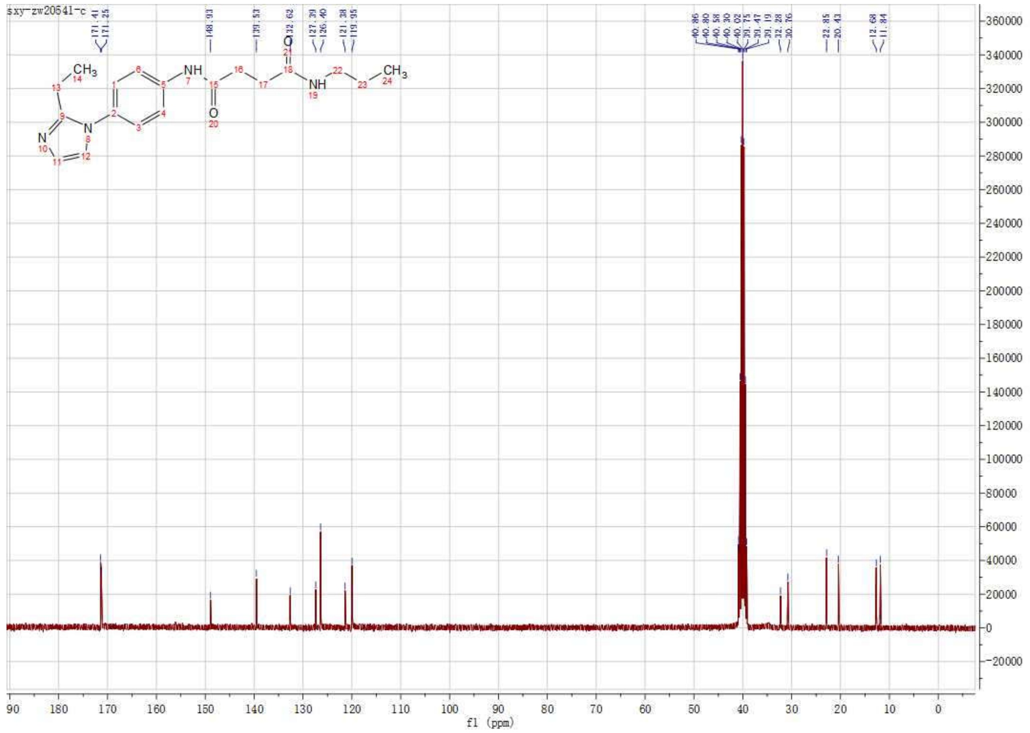

HIGH RESLUTION MASS SPECTROMETRY of Compound **4a**

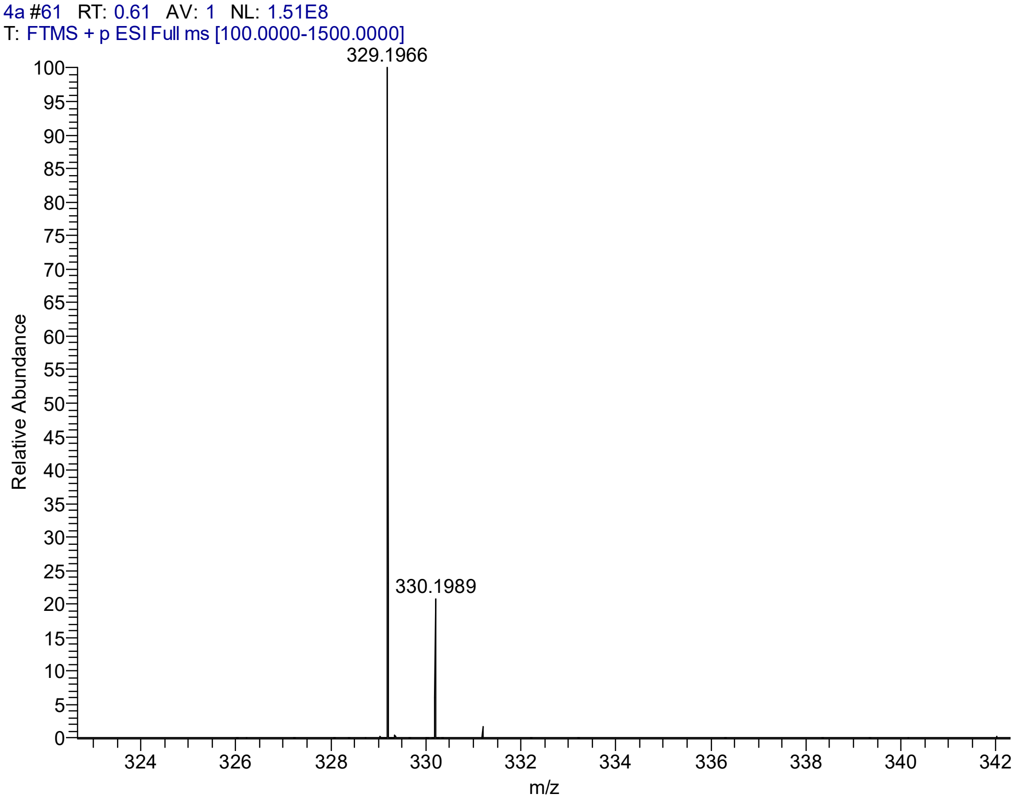

^{1}H-NMR spectrum of Compound **4b**

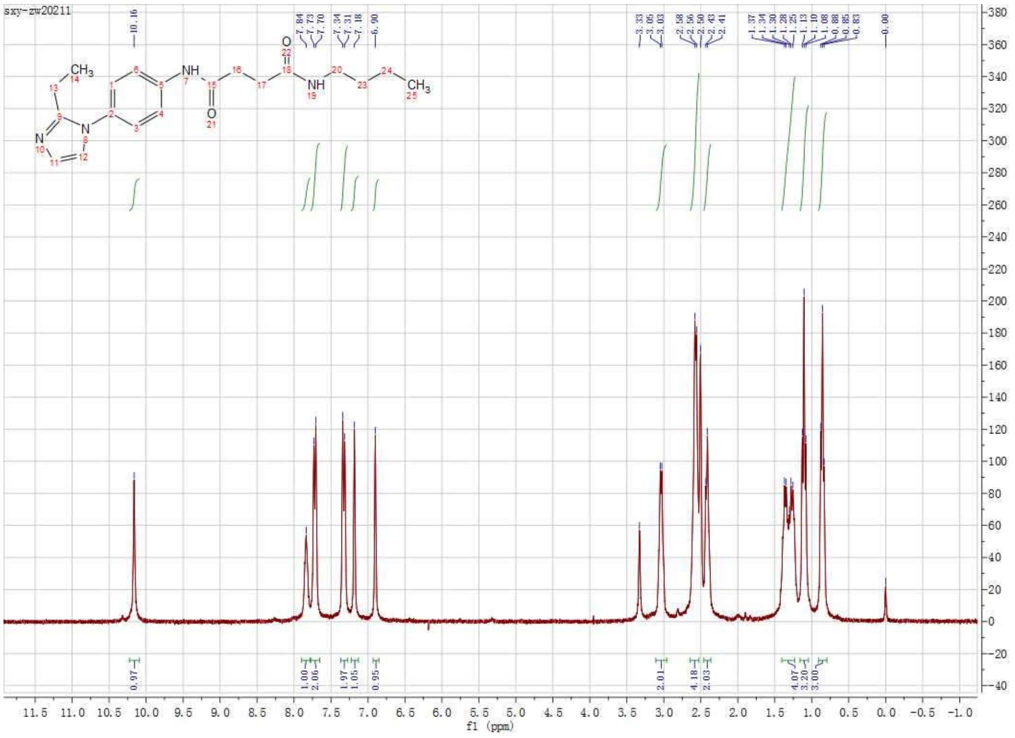

^{13}C-NMR spectrum of Compound **4b**

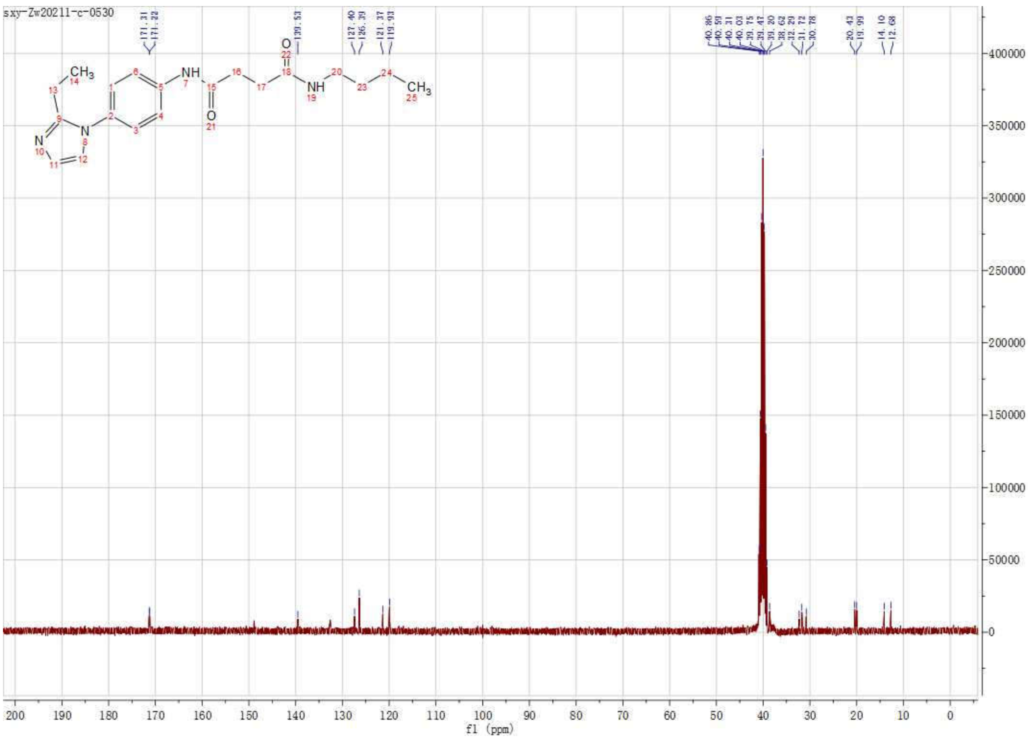

HIGH RESLUTION MASS SPECTROMETRY of Compound **4b**

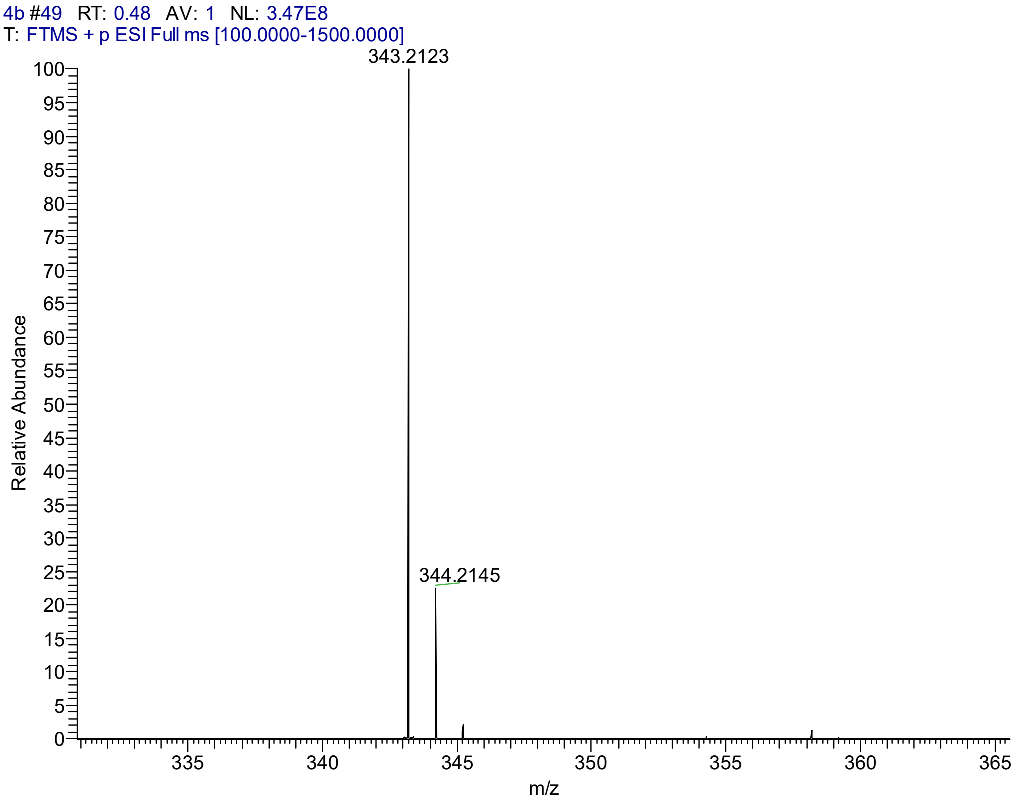

^{1}H-NMR spectrum of Compound **4c**

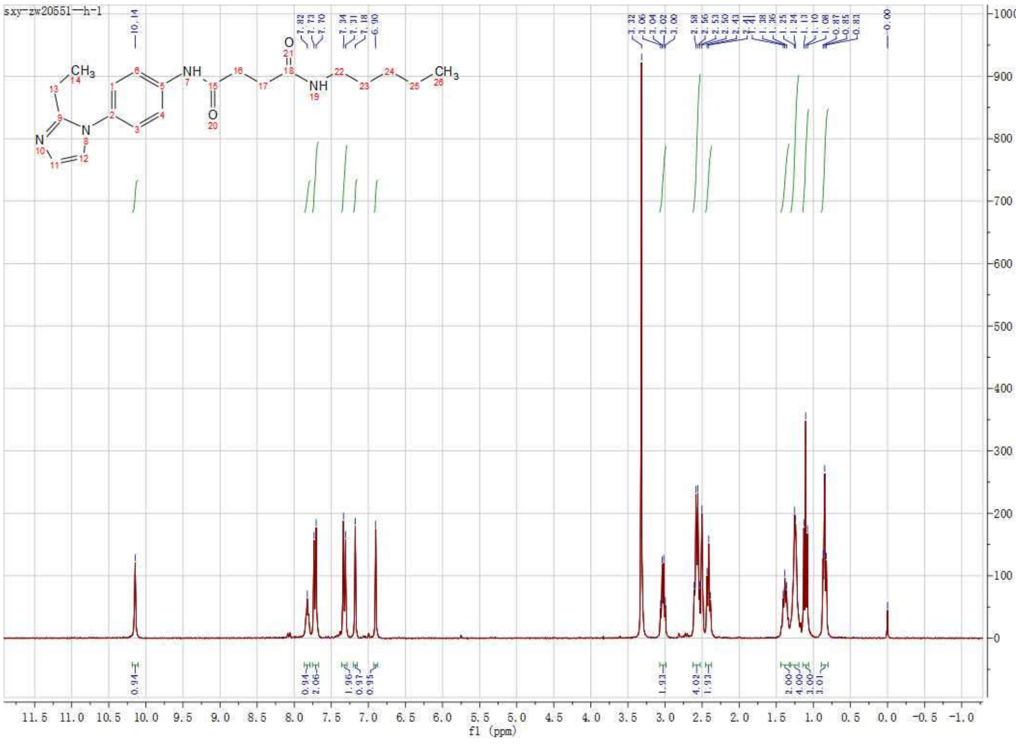

^{13}C-NMR spectrum of Compound **4c**

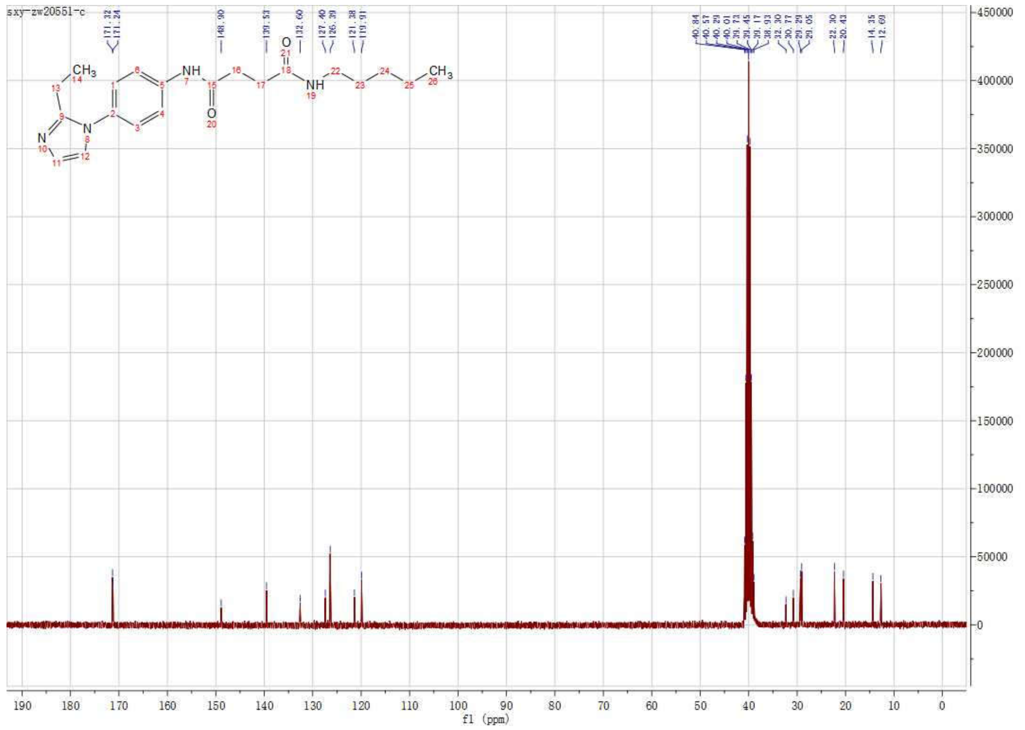

HIGH RESLUTION MASS SPECTROMETRY of Compound **4c**

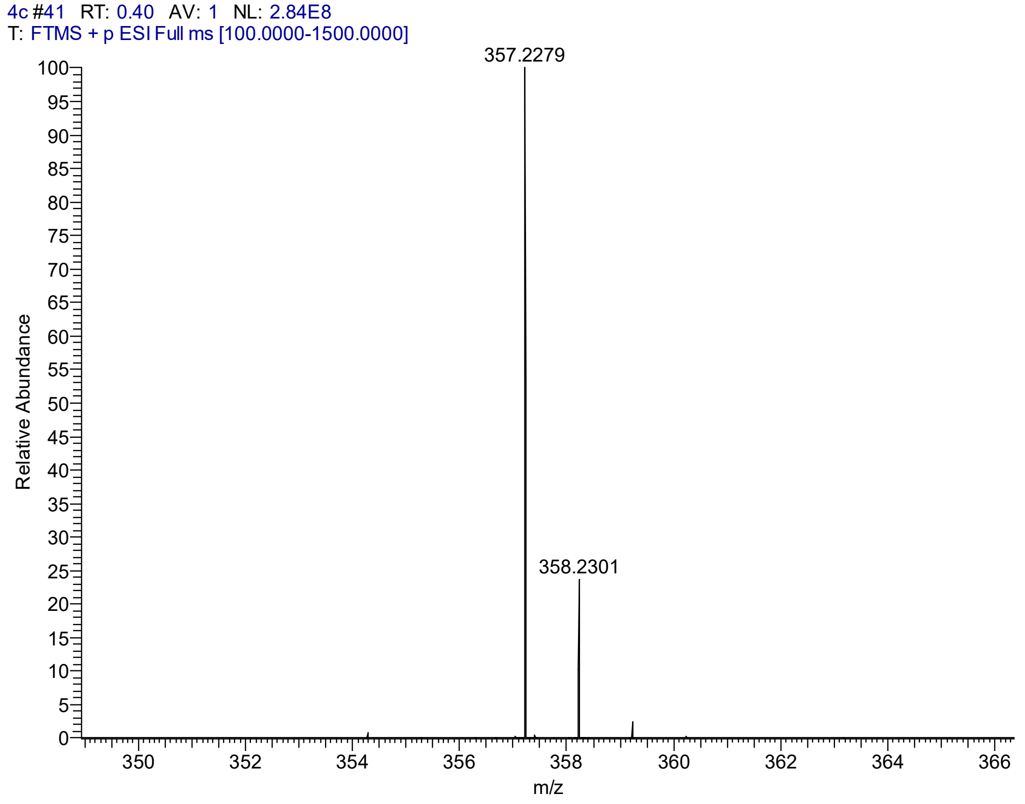

^{1}H-NMR spectrum of Compound **4d**

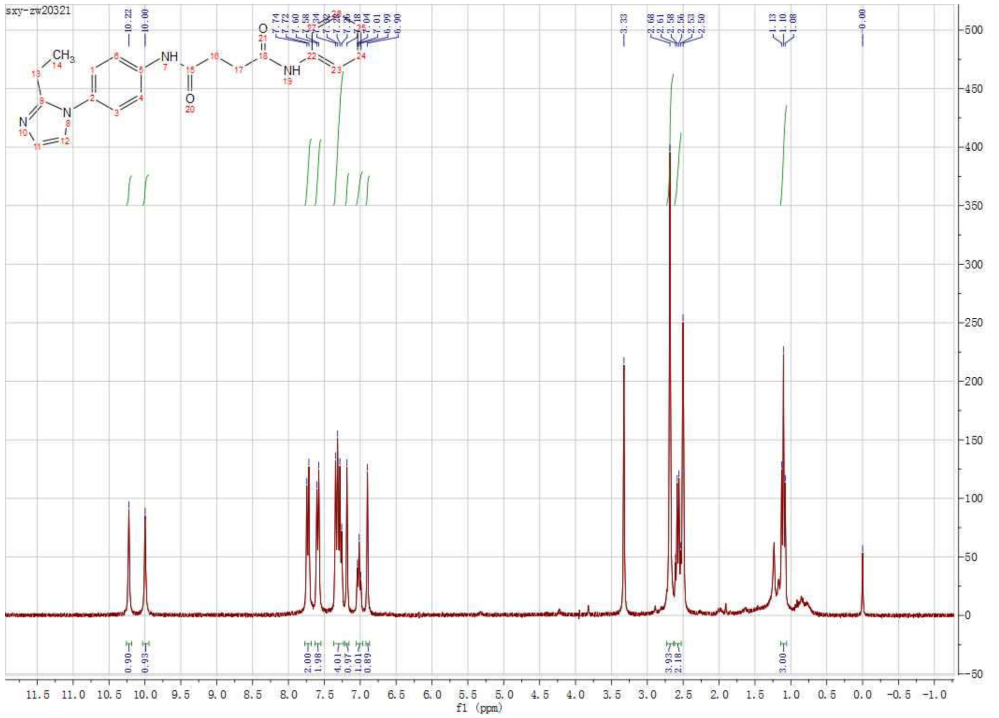

^{13}C-NMR spectrum of Compound **4d**

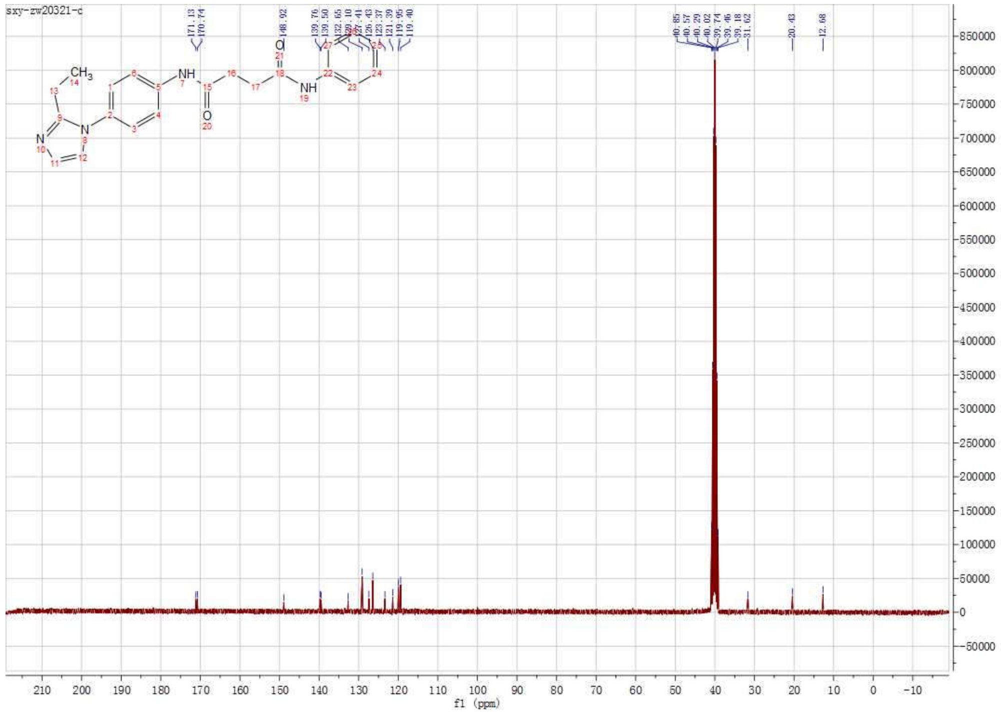

HIGH RESLUTION MASS SPECTROMETRY of Compound **4d**

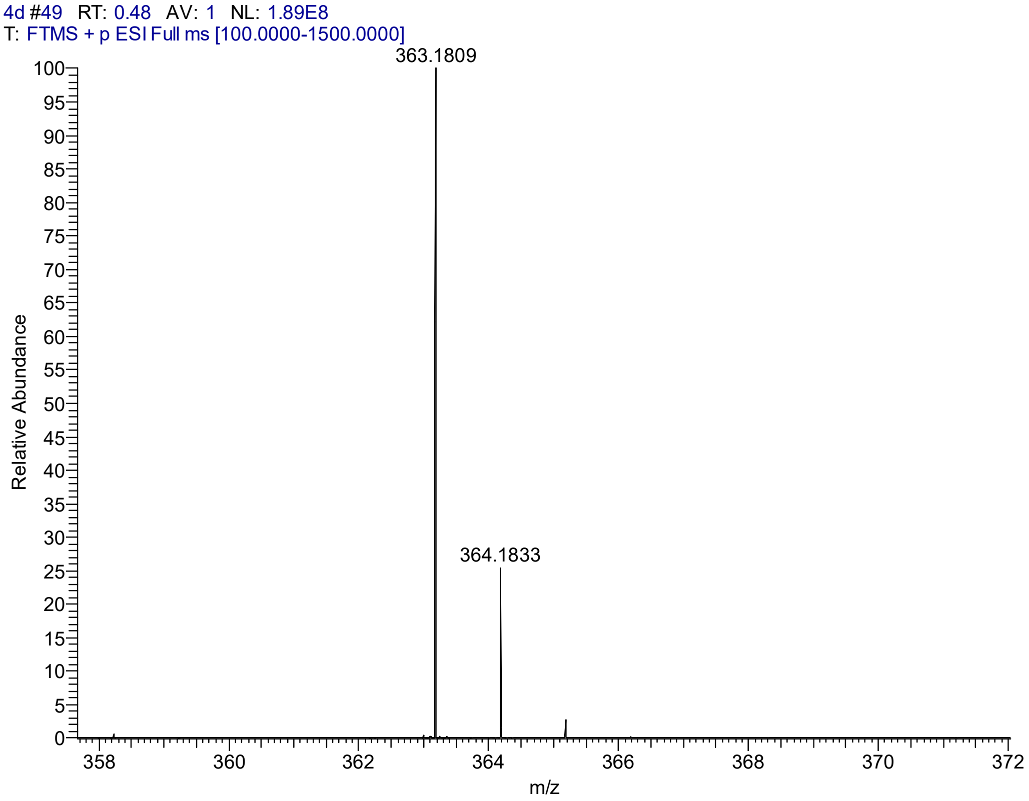

^{1}H-NMR spectrum of Compound **4e**

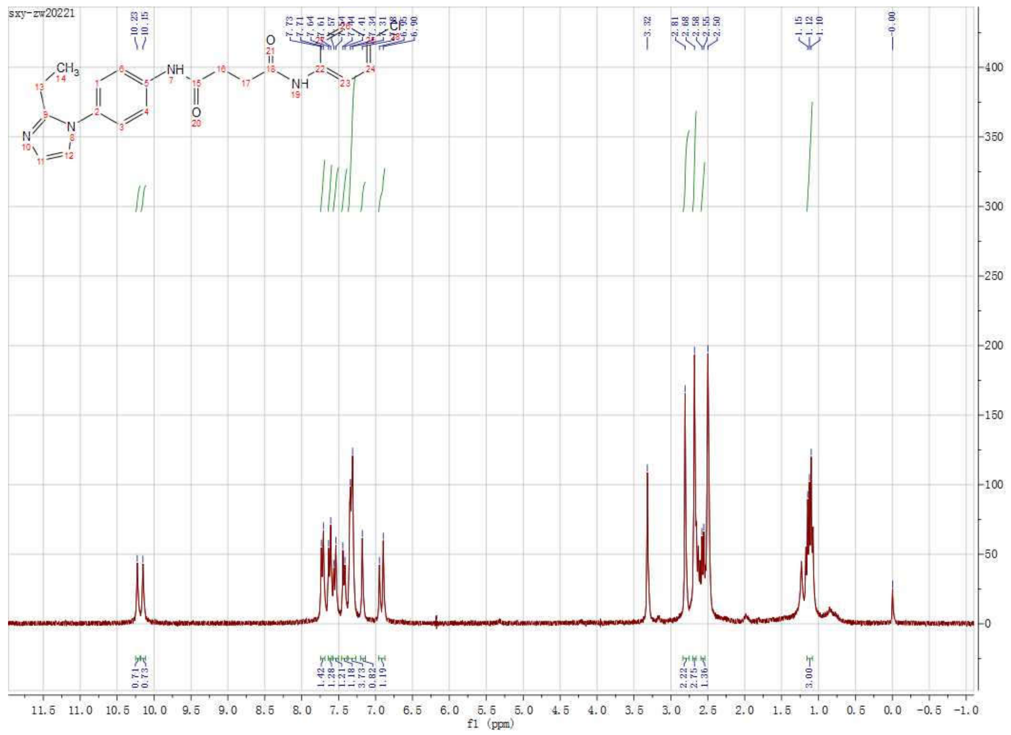

^{13}C-NMR spectrum of Compound **4e**

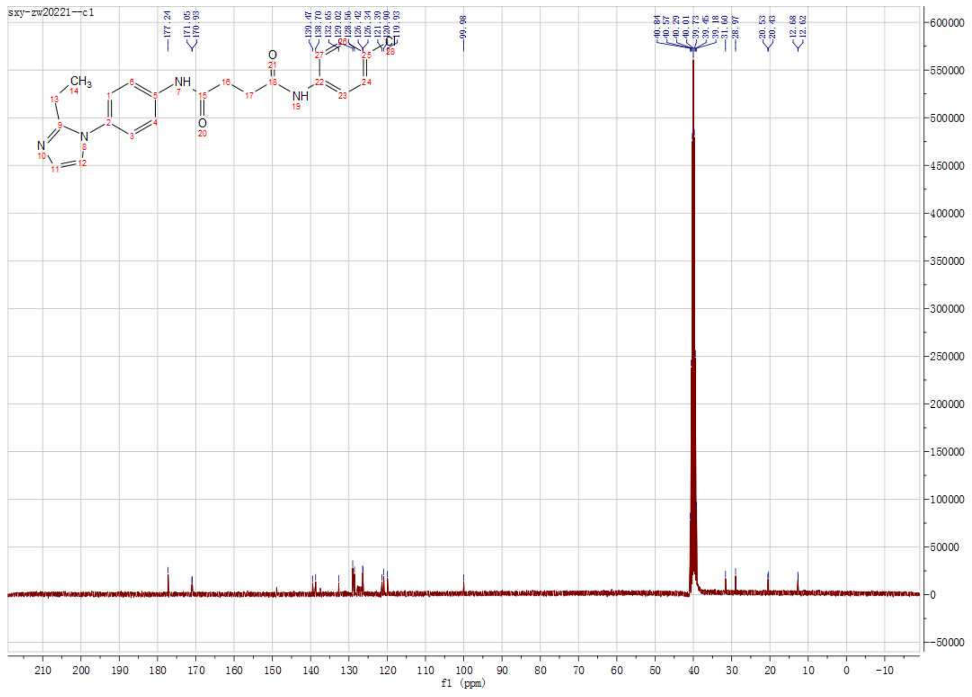

HIGH RESLUTION MASS SPECTROMETRY of Compound **4e**

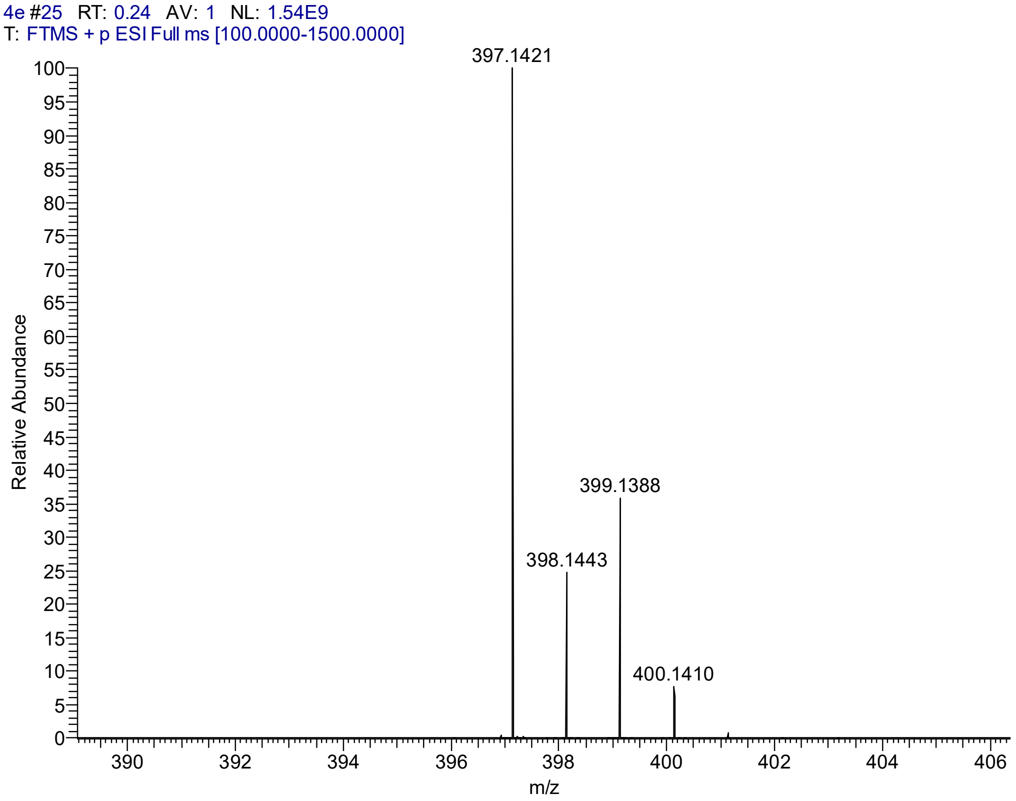

^{1}H-NMR spectrum of Compound **4f**

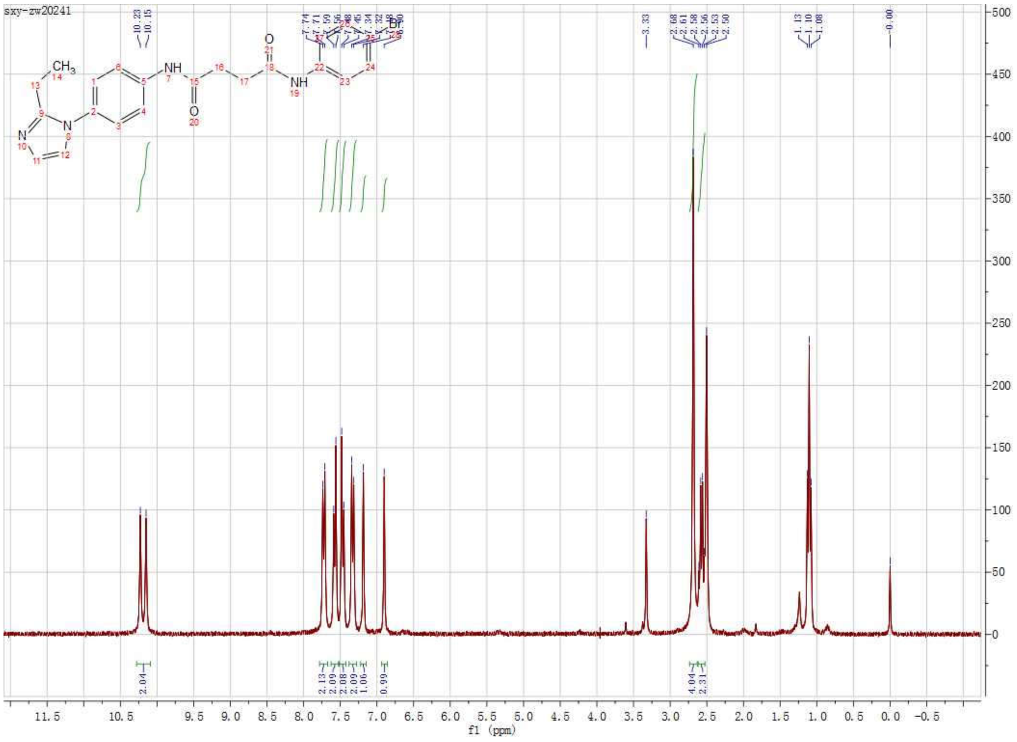

^{13}C-NMR spectrum of Compound **4f**

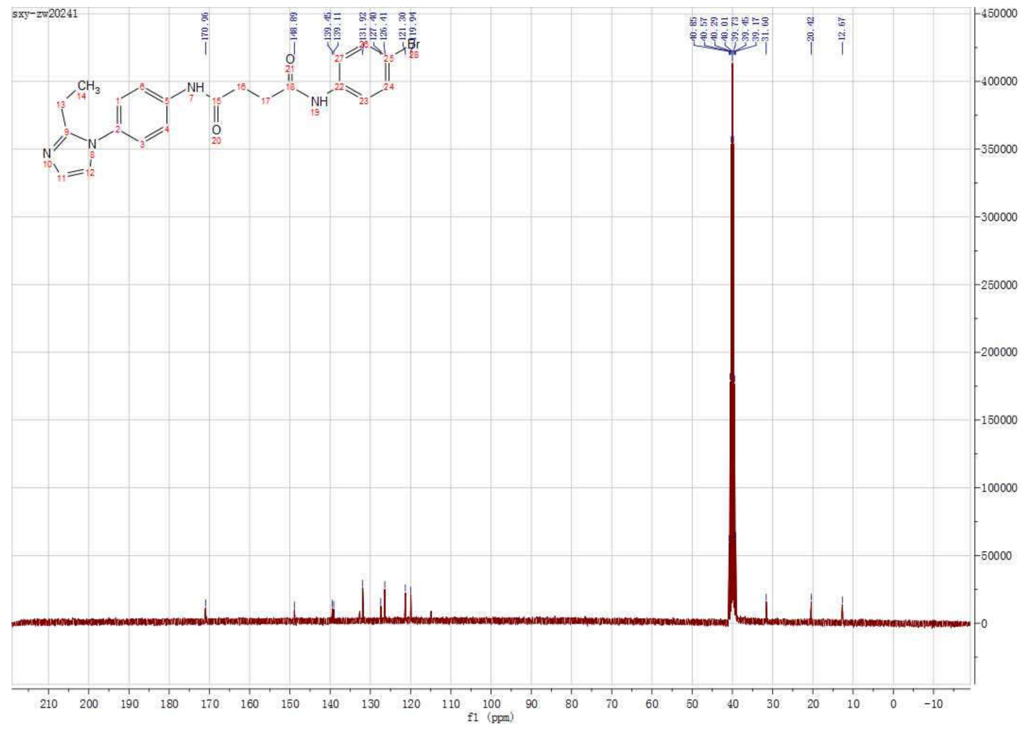

HIGH RESLUTION MASS SPECTROMETRY of Compound **4f**

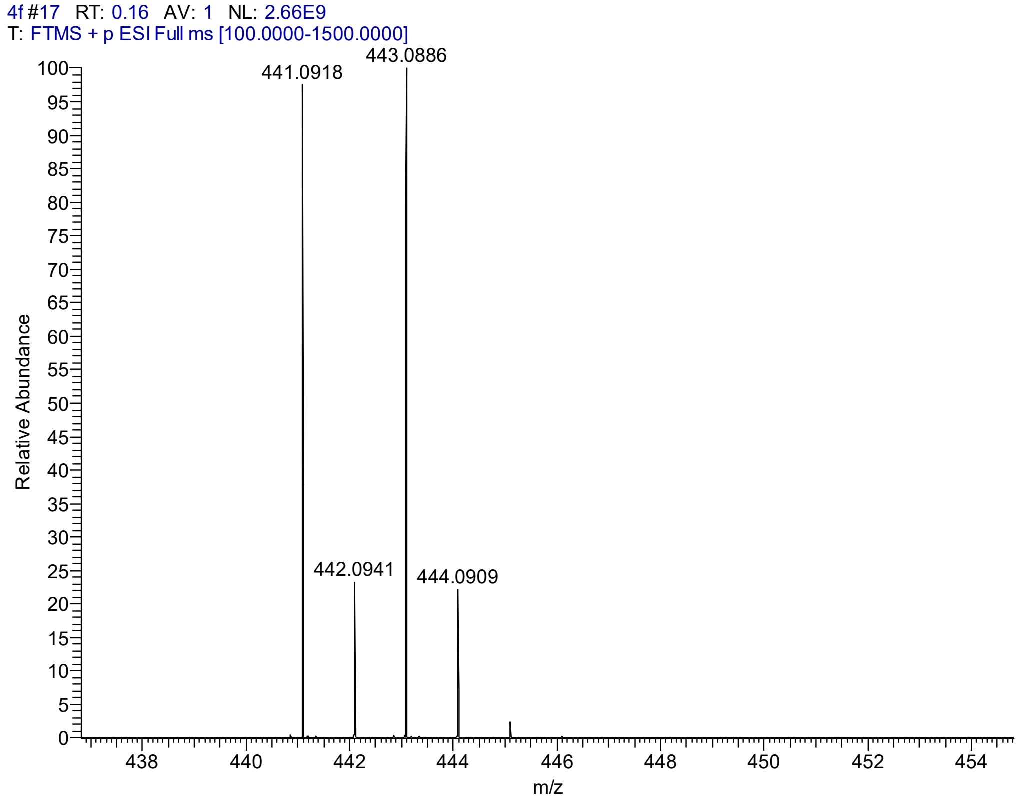

^{1}H-NMR spectrum of Compound **4g**

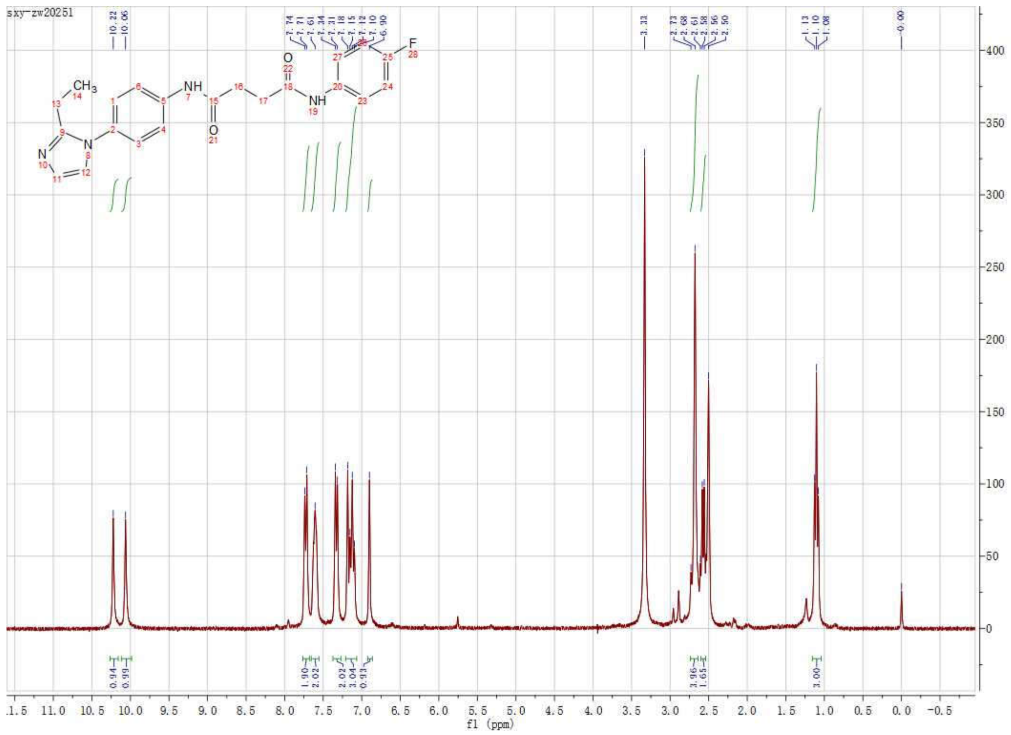

^{13}C-NMR spectrum of Compound **4g**

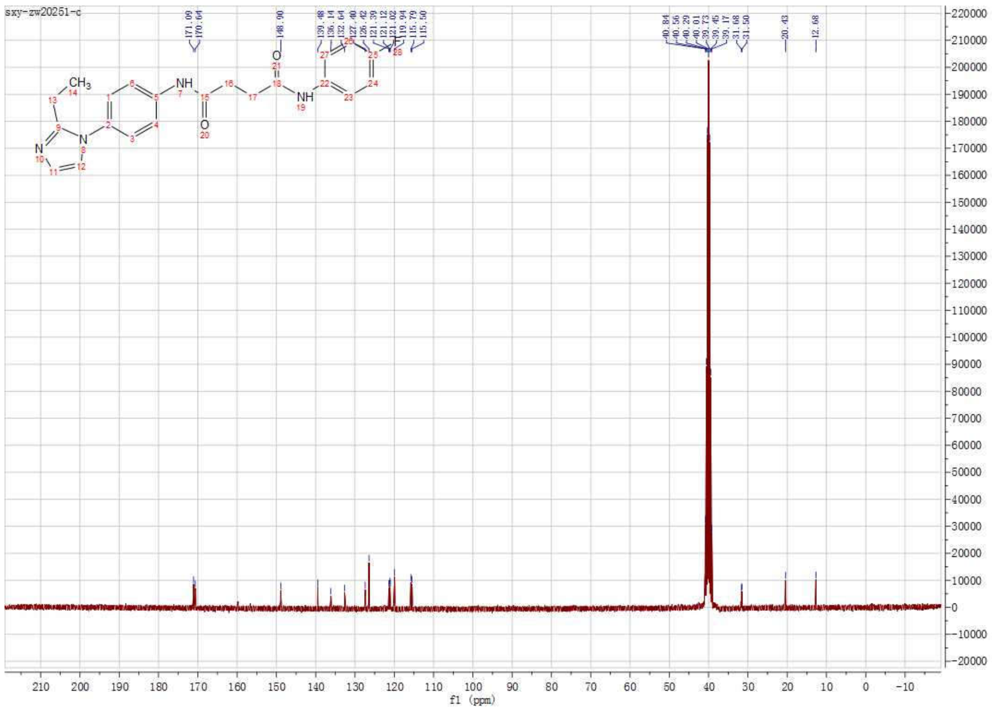

HIGH RESLUTION MASS SPECTROMETRY of Compound **4g**

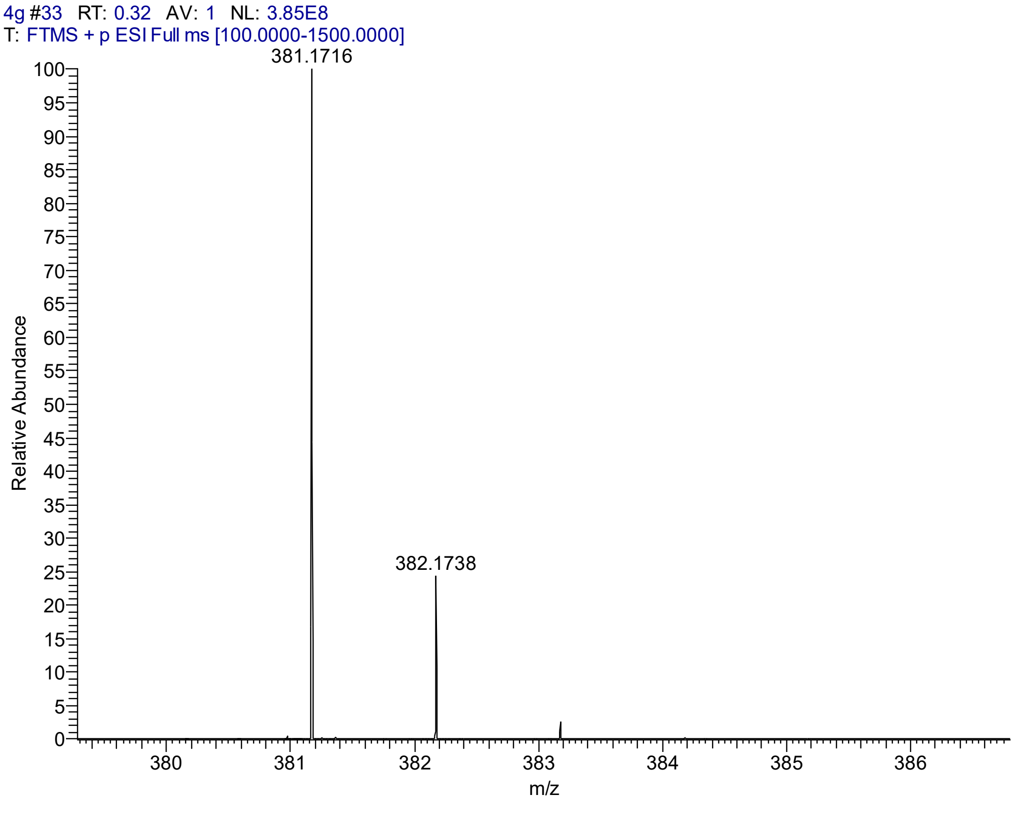

^{1}H-NMR spectrum of Compound **4h**

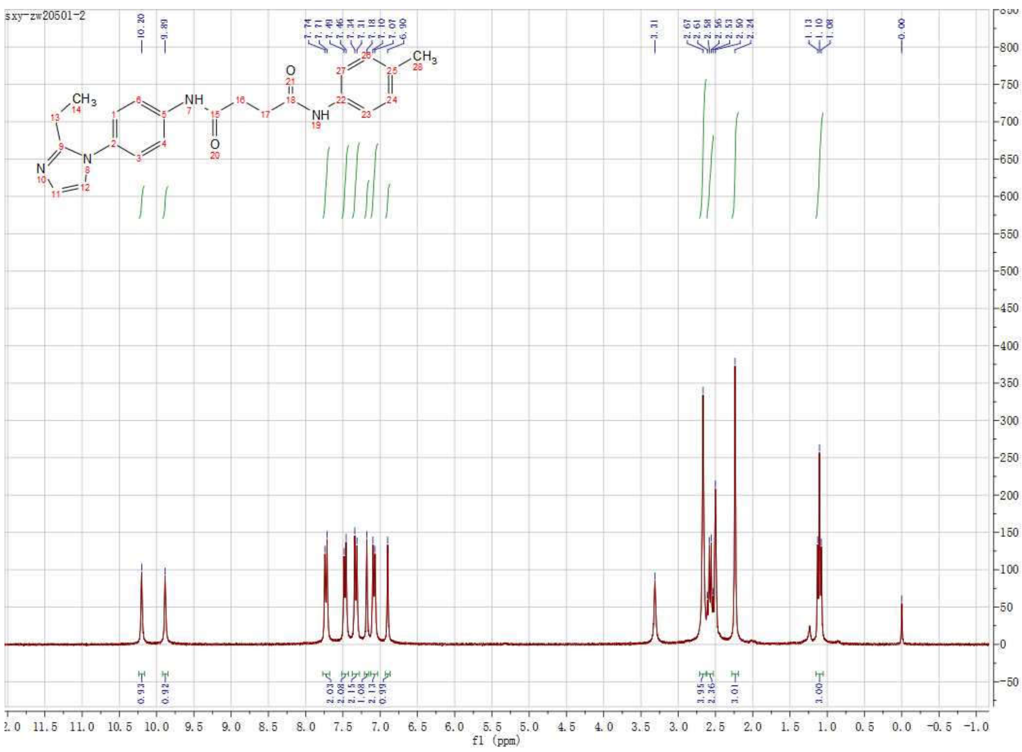

^{13}C-NMR spectrum of Compound **4h**

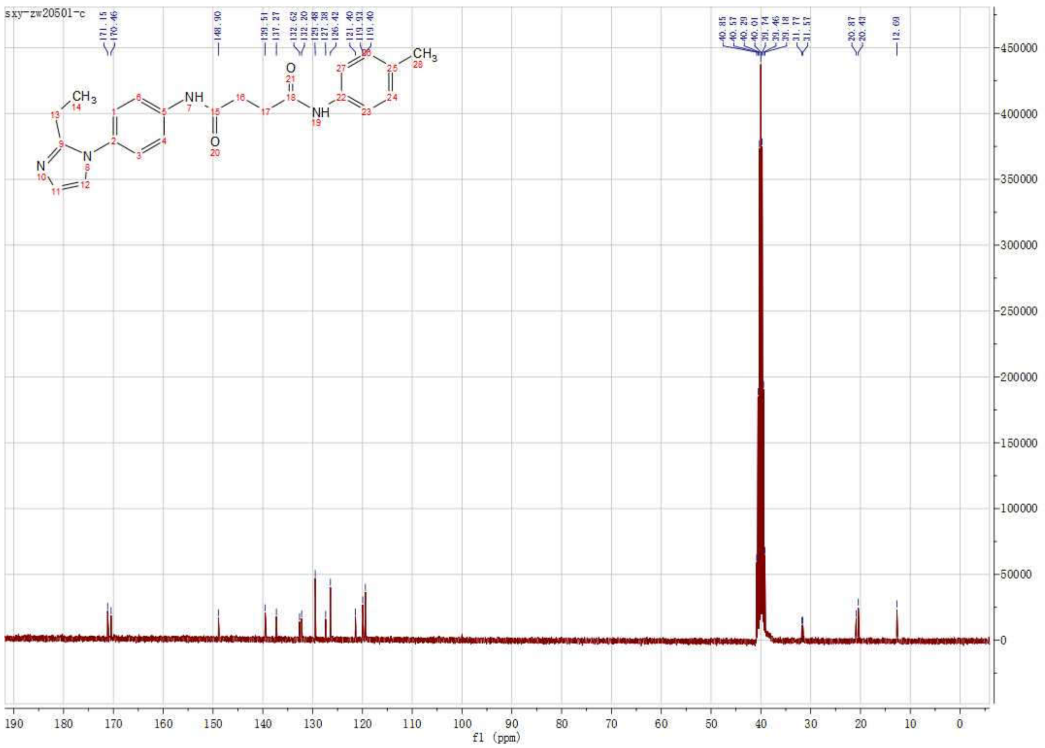

HIGH RESLUTION MASS SPECTROMETRY of Compound **4h**

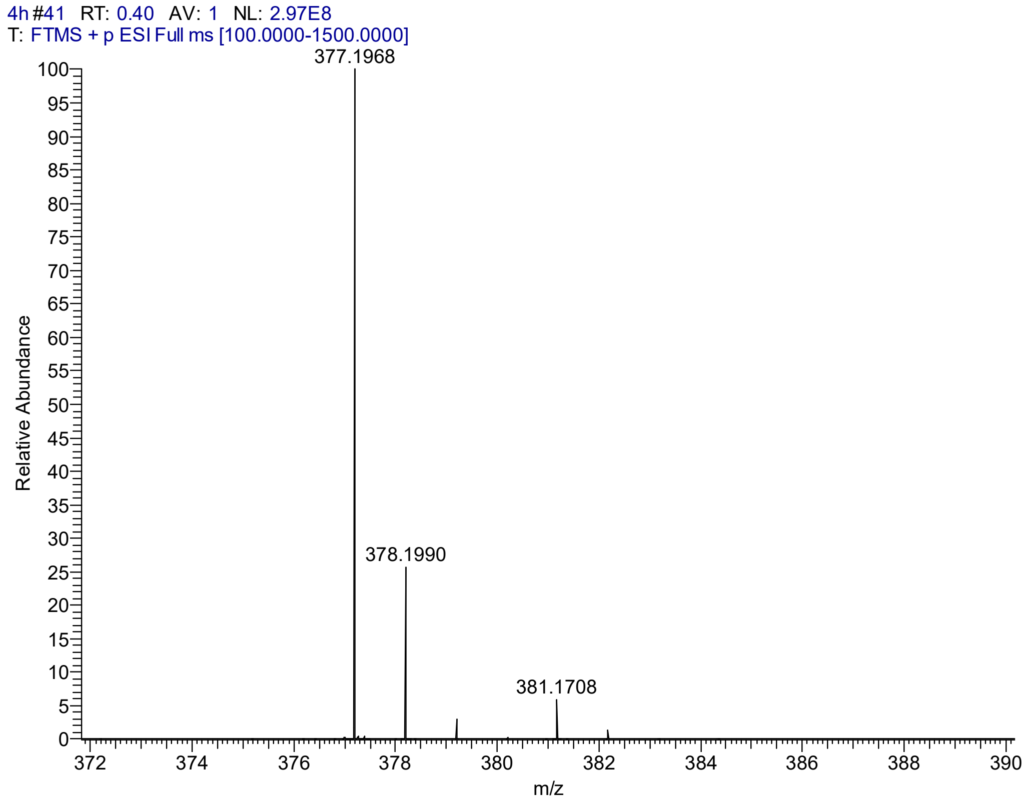

^{1}H-NMR spectrum of Compound **4i**

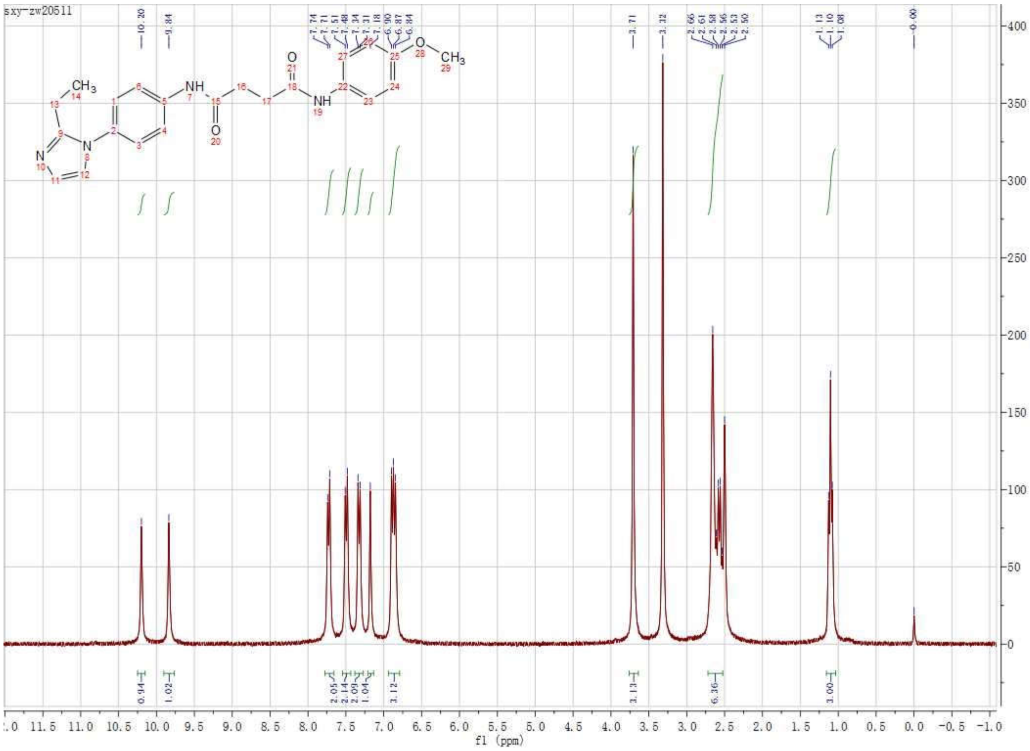

^{13}C-NMR spectrum of Compound **4i**

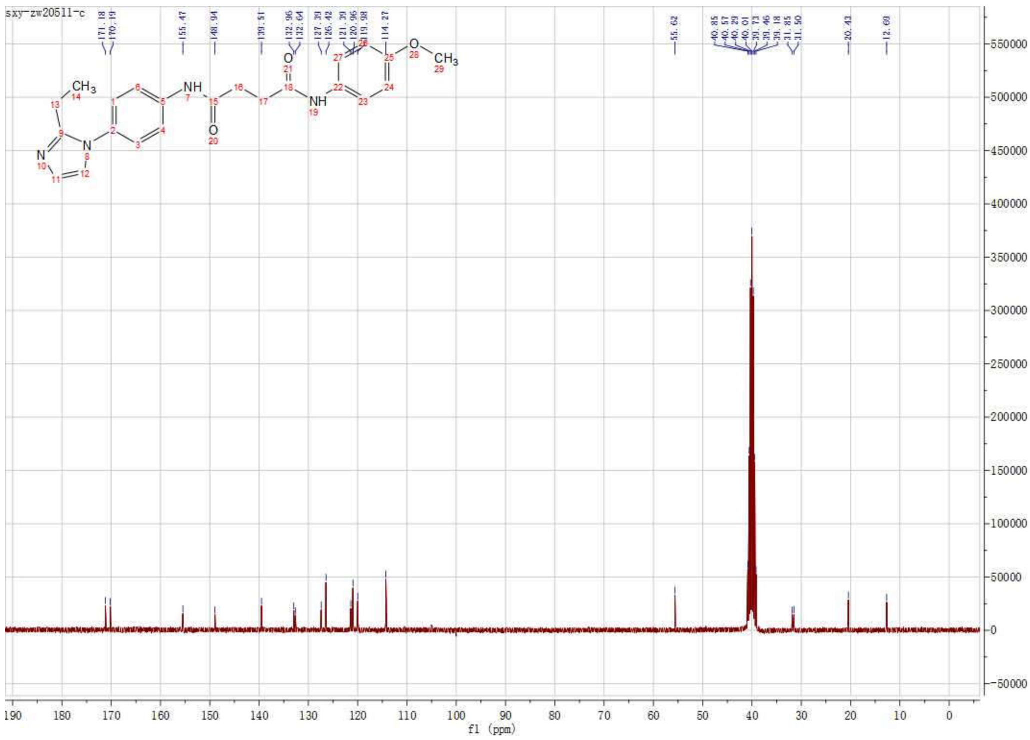

HIGH RESLUTION MASS SPECTROMETRY of Compound **4i**

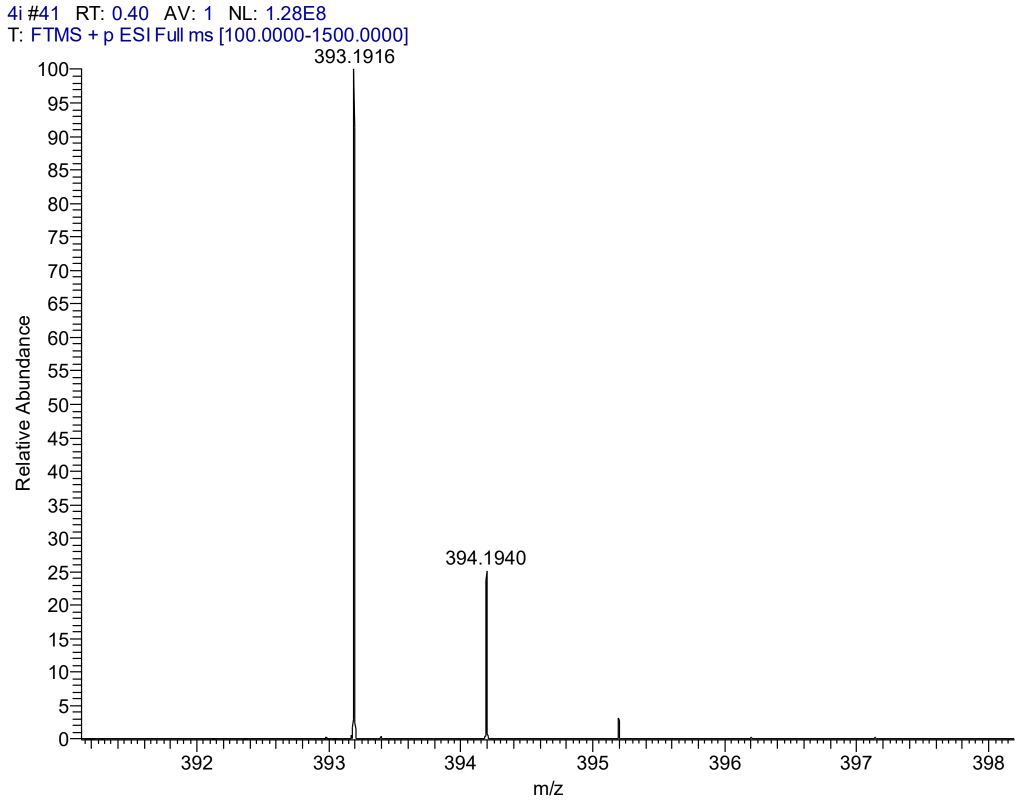

^{1}H-NMR spectrum of Compound **4j**

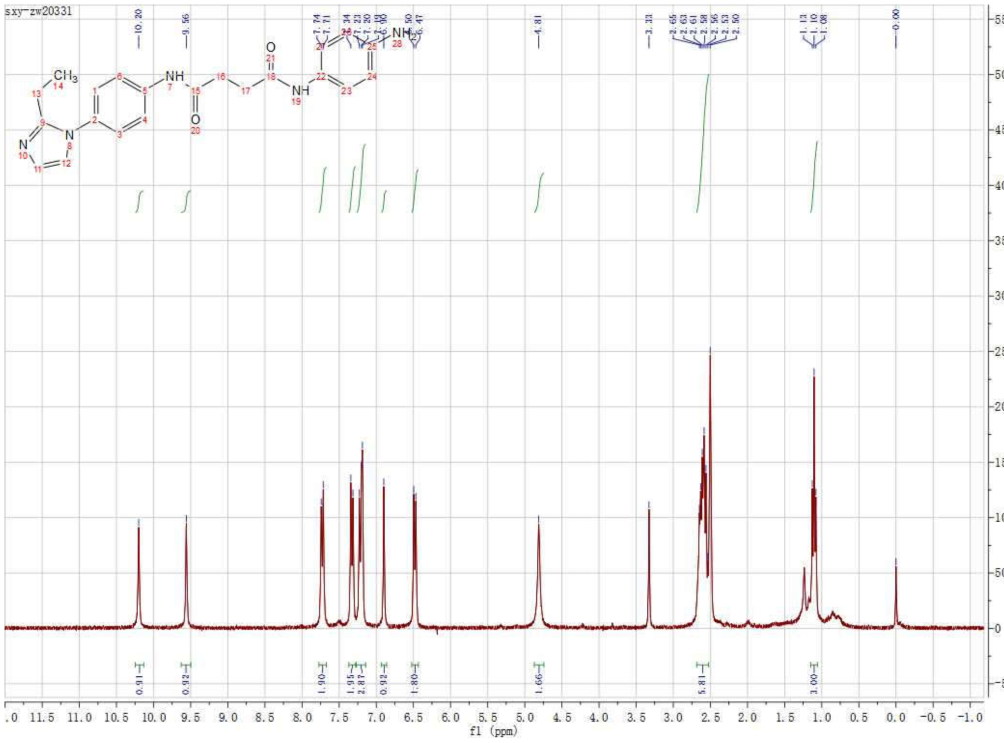

^{13}C-NMR spectrum of Compound **4j**

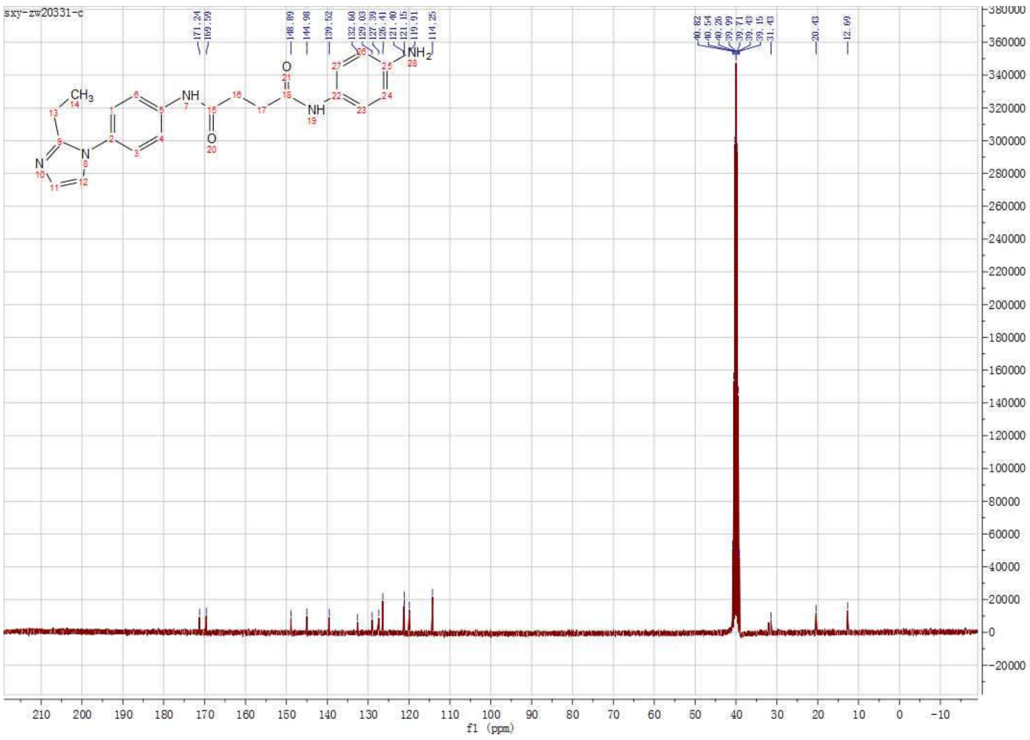

HIGH RESLUTION MASS SPECTROMETRY of Compound **4j**

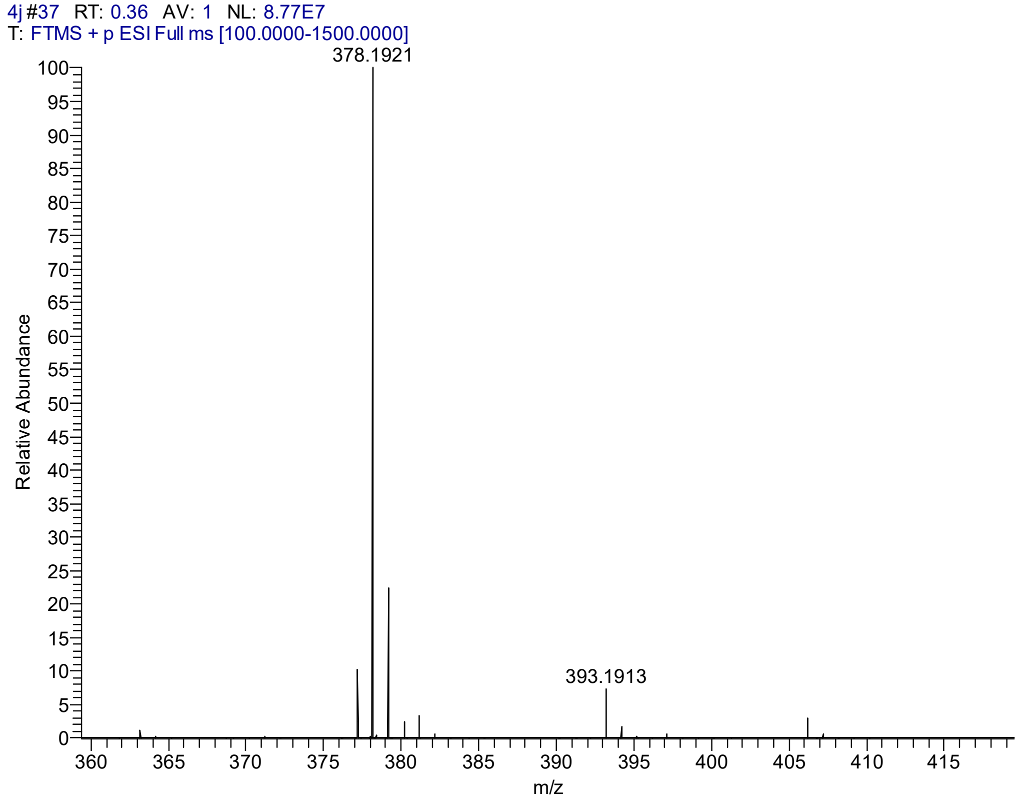

^{1}H-NMR spectrum of Compound **4k**

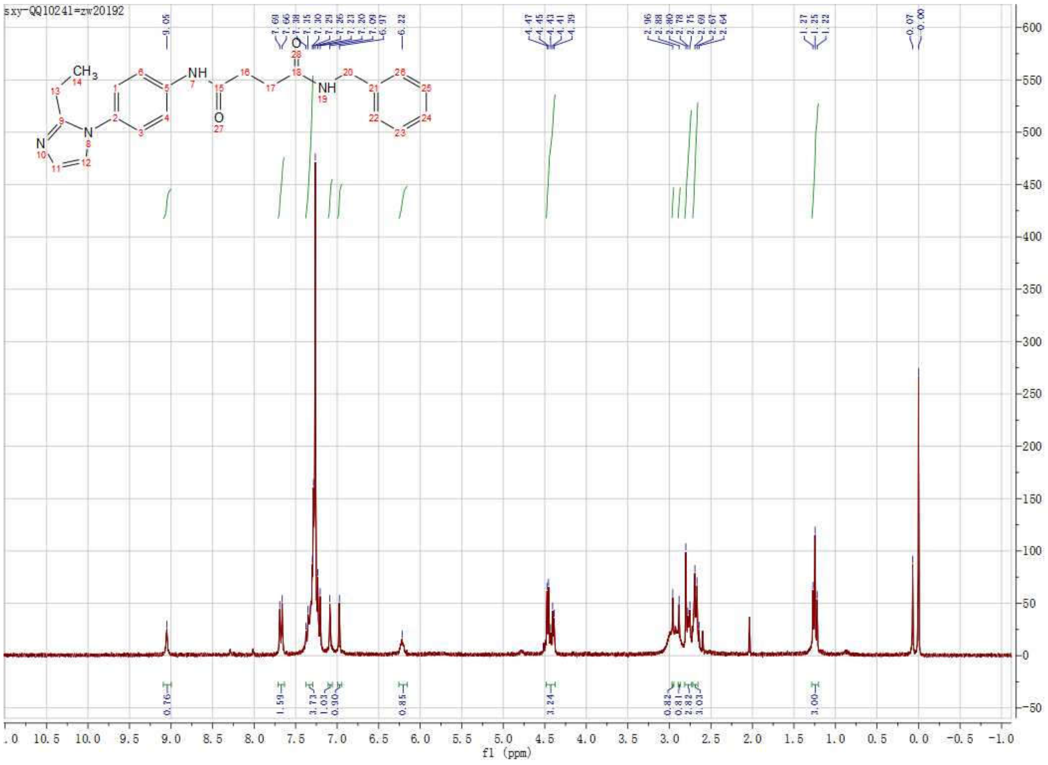

^{13}C-NMR spectrum of Compound **4k**

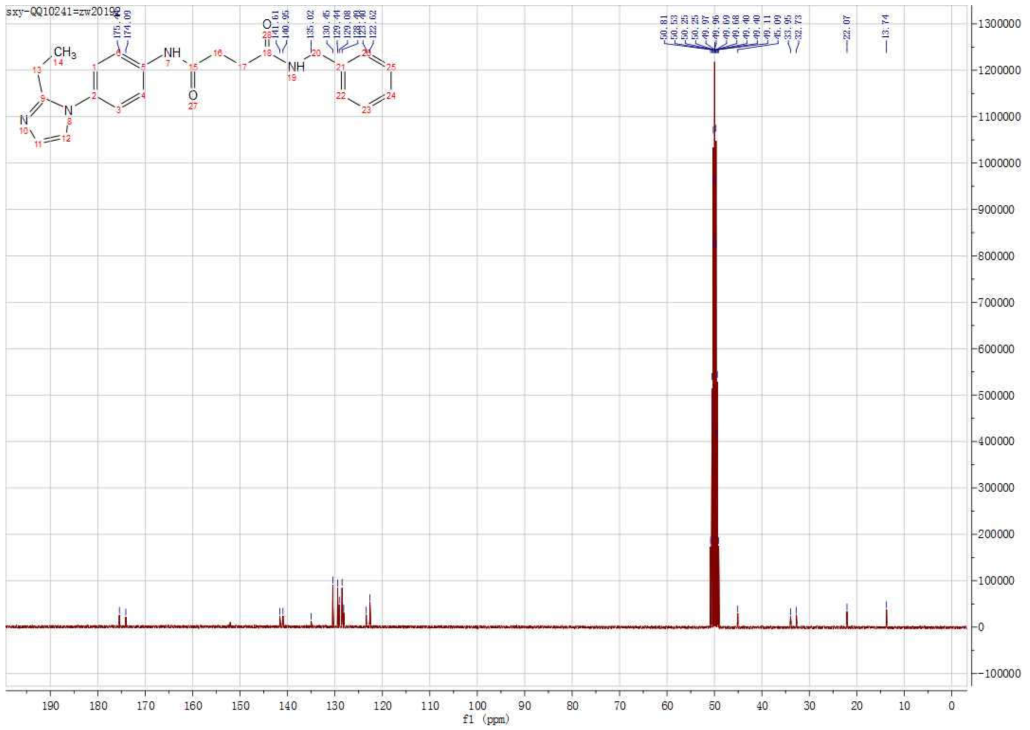

HIGH RESLUTION MASS SPECTROMETRY of Compound **4k**

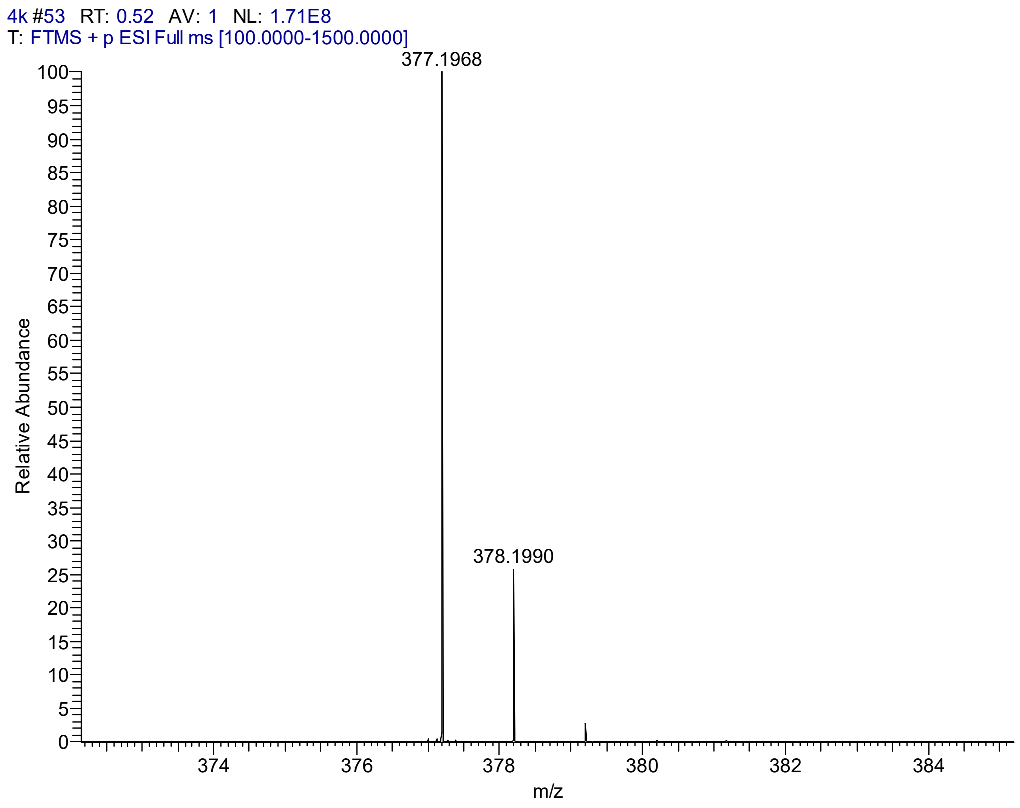

^{1}H-NMR spectrum of Compound **4l**

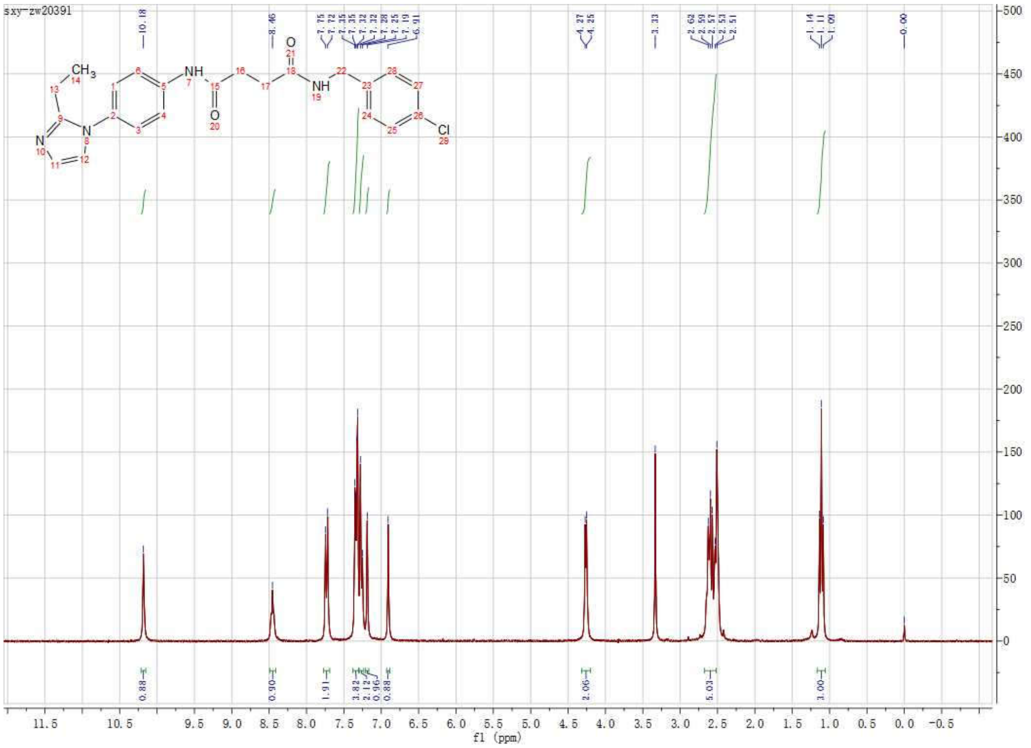

^{13}C-NMR spectrum of Compound **4l**

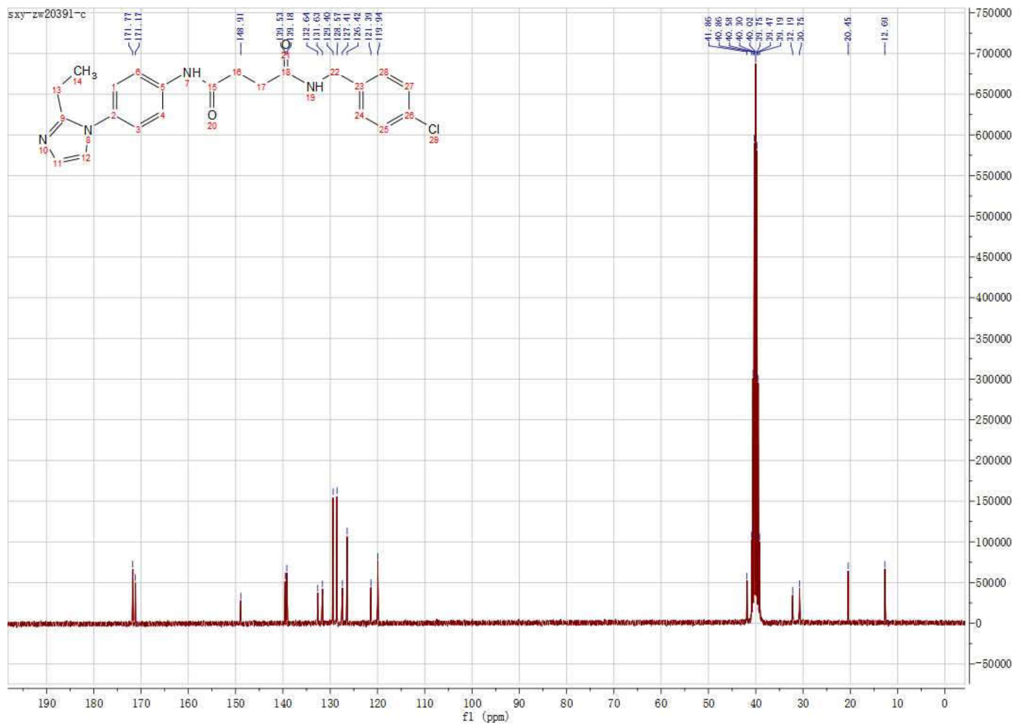

HIGH RESLUTION MASS SPECTROMETRY of Compound **4l**

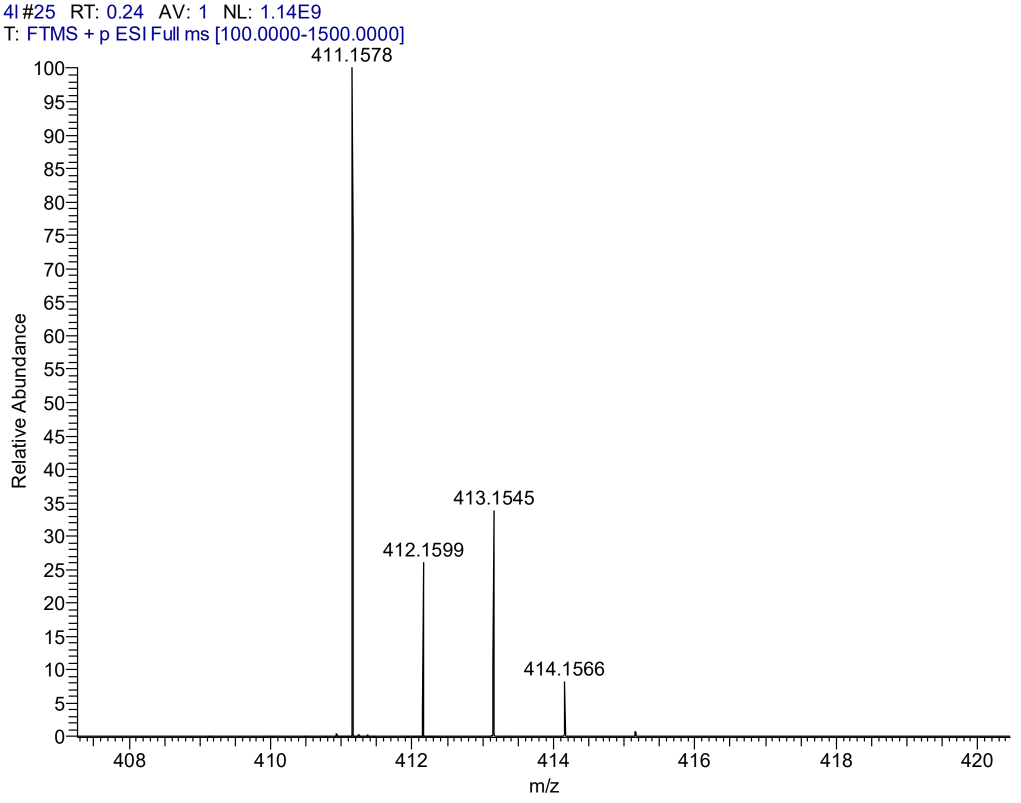

^{1}H-NMR spectrum of Compound **4m**

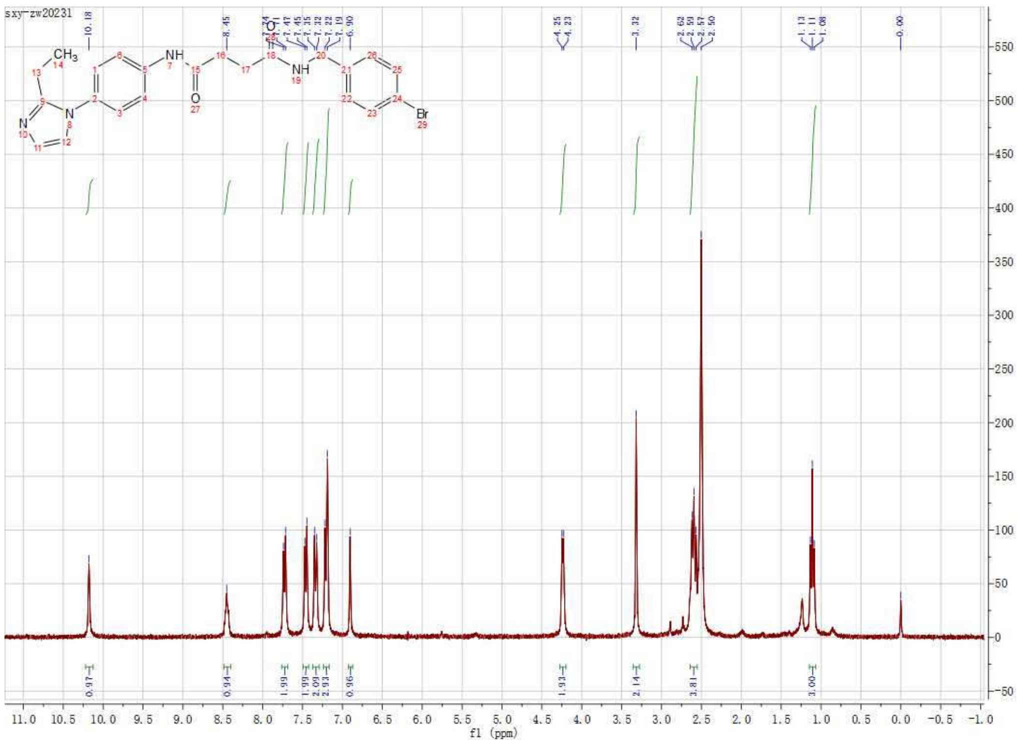

^{13}C-NMR spectrum of Compound **4m**

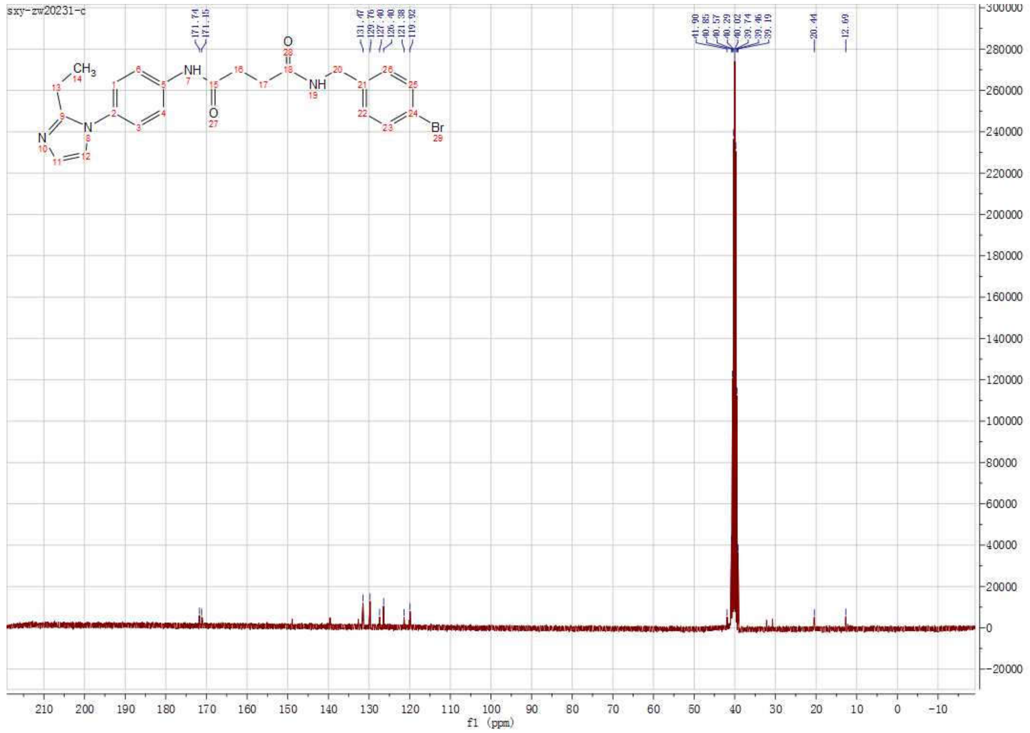

HIGH RESLUTION MASS SPECTROMETRY of Compound **4m**

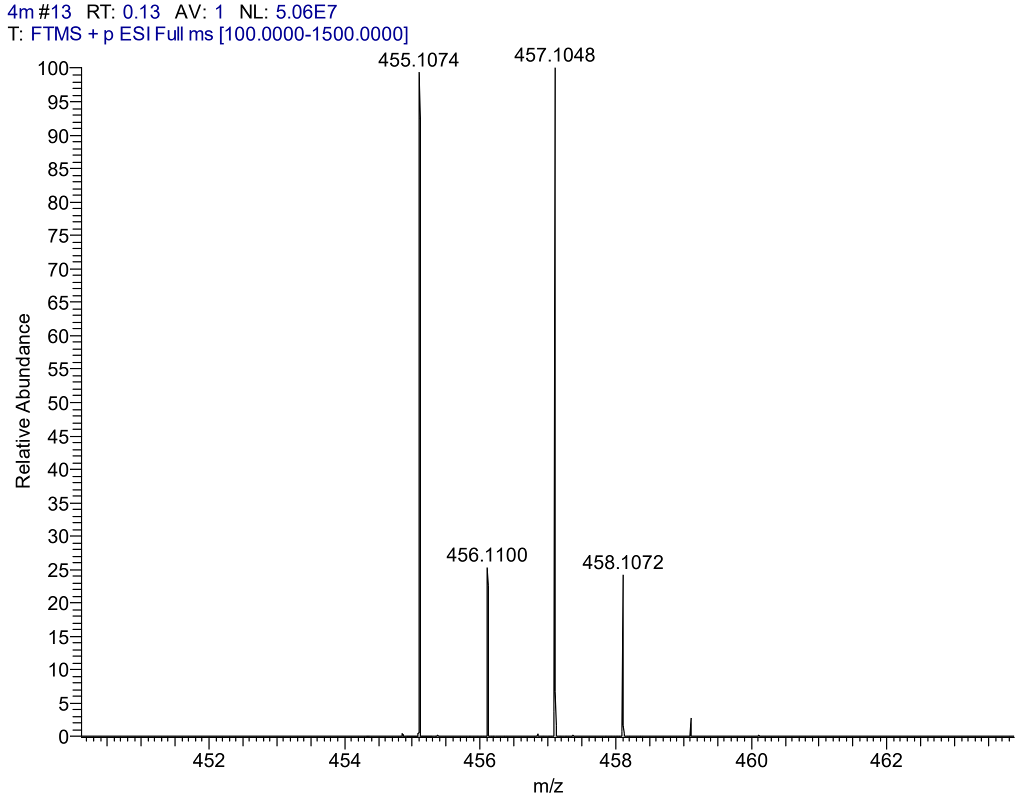

^{1}H-NMR spectrum of Compound **4n**

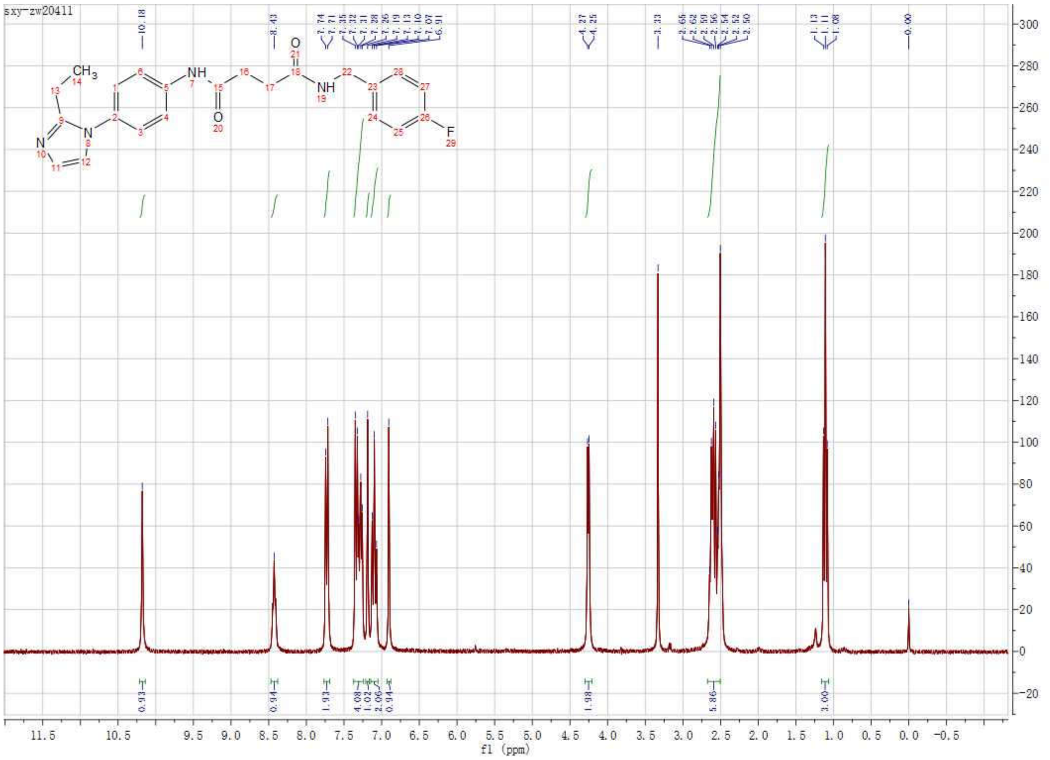

^{13}C-NMR spectrum of Compound **4n**

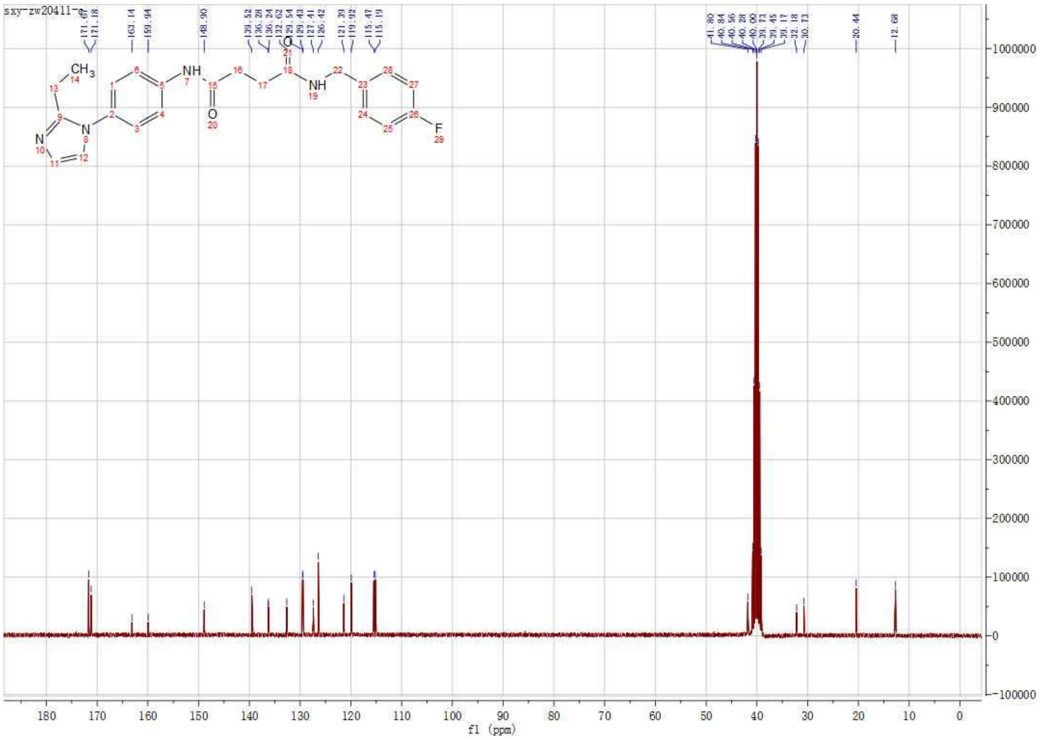

HIGH RESLUTION MASS SPECTROMETRY of Compound **4n**

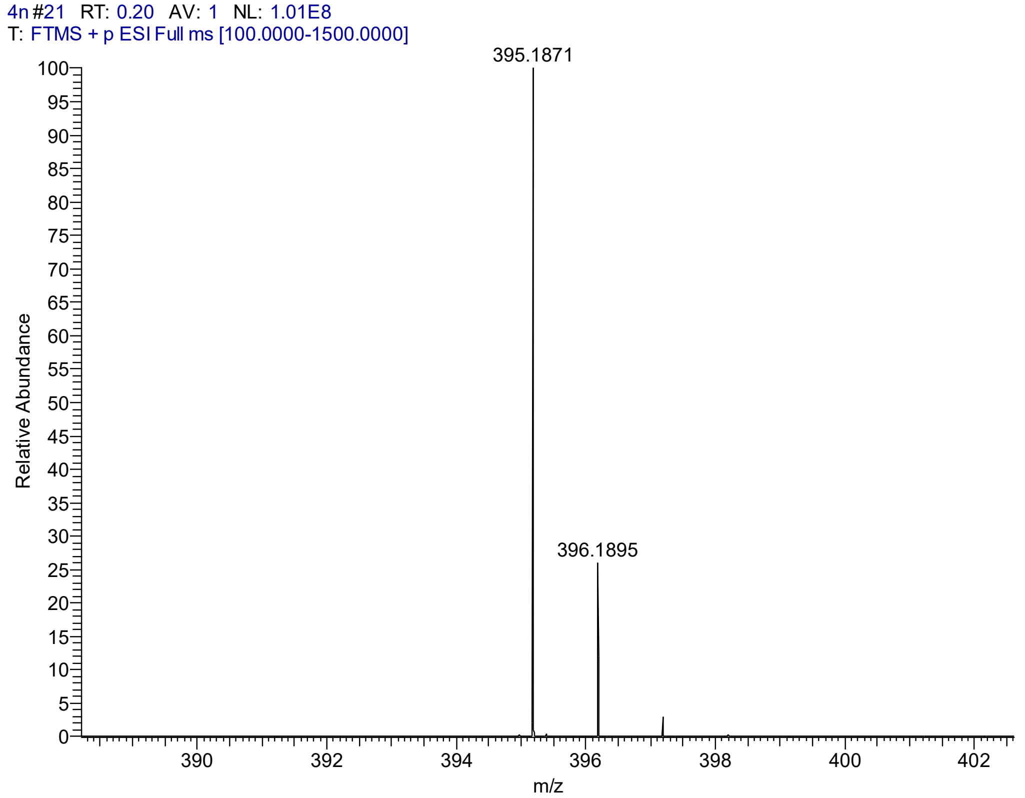

^{1}H-NMR spectrum of Compound **4o**

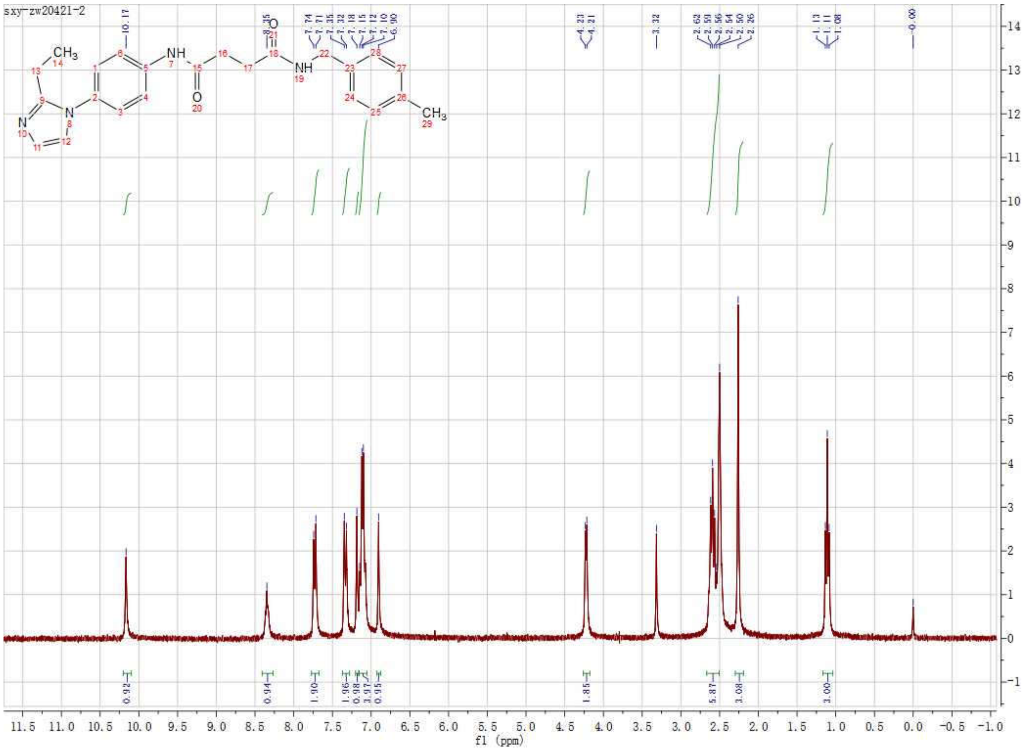

^{13}C-NMR spectrum of Compound **4o**

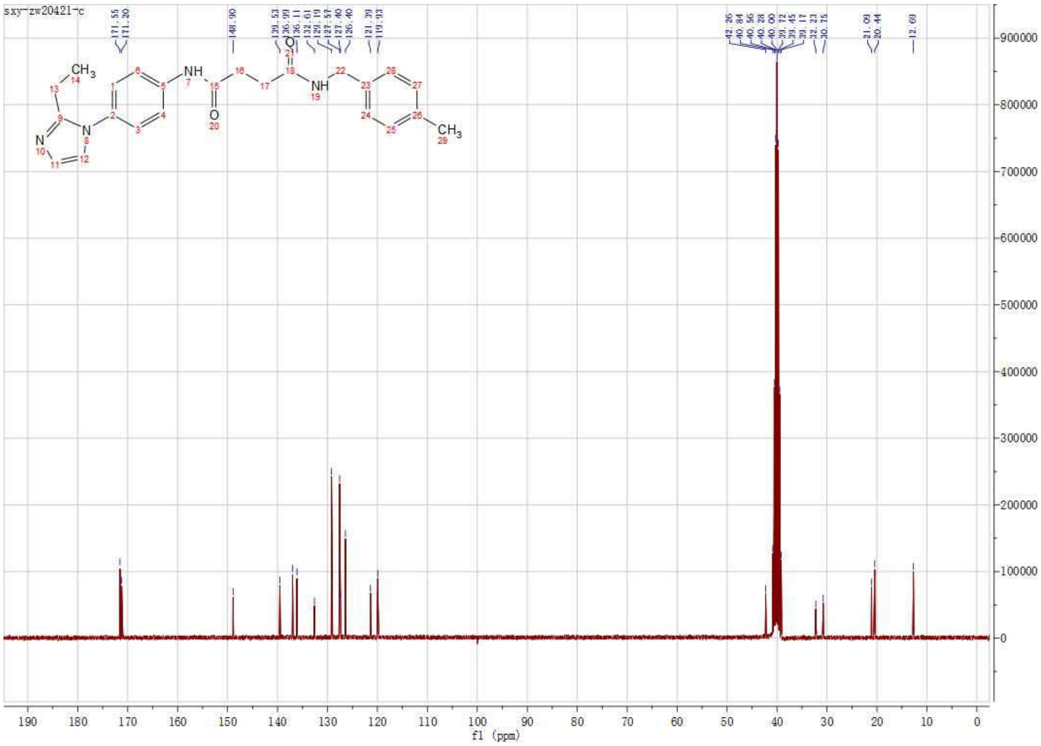

sxy-zw20421-c
171.55
171.20
148.90
139.53
136.99
136.11
132.61
129.19
127.57
127.40
126.40
121.39
119.93
42.26
40.84
40.56
40.28
40.00
39.72
39.45
39.17
32.23
30.75
21.09
20.44
12.69
CH3
NH
O
N
f1 (ppm)
190
180
170
160
150
140
130
120
110
100
90
80
70
60
50
40
30
20
10
0
900000
800000
700000
600000
500000
400000
300000
200000
100000
0

HIGH RESLUTION MASS SPECTROMETRY of Compound **4o**

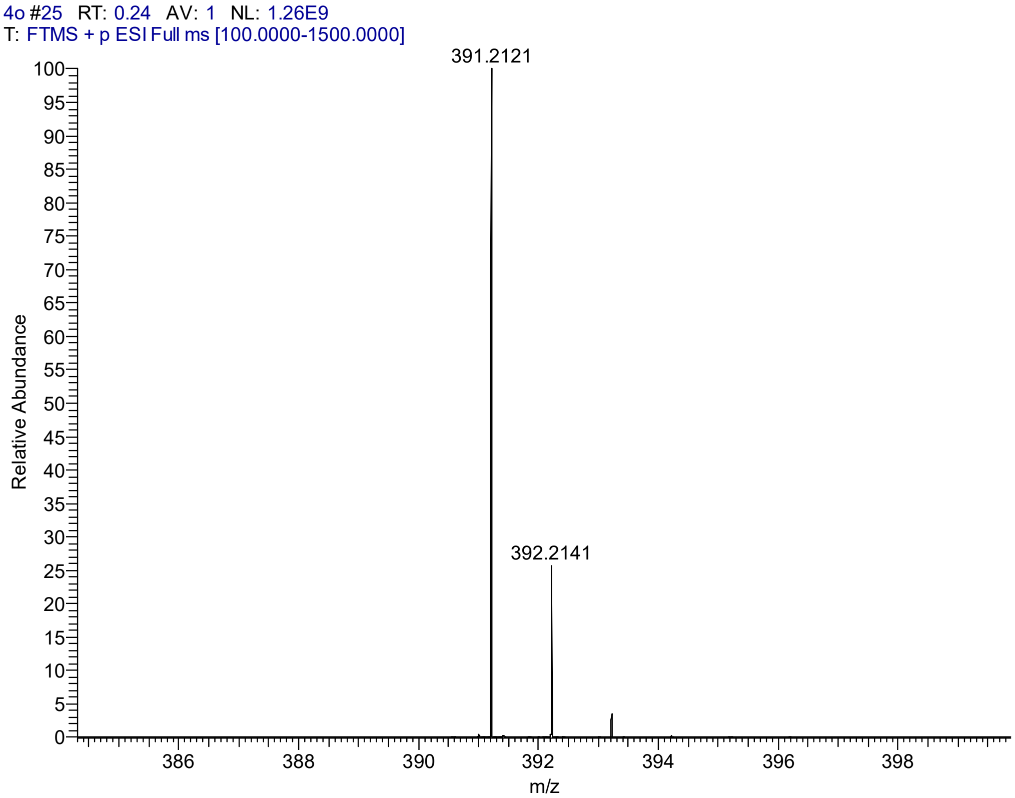

^{1}H-NMR spectrum of Compound **4p**

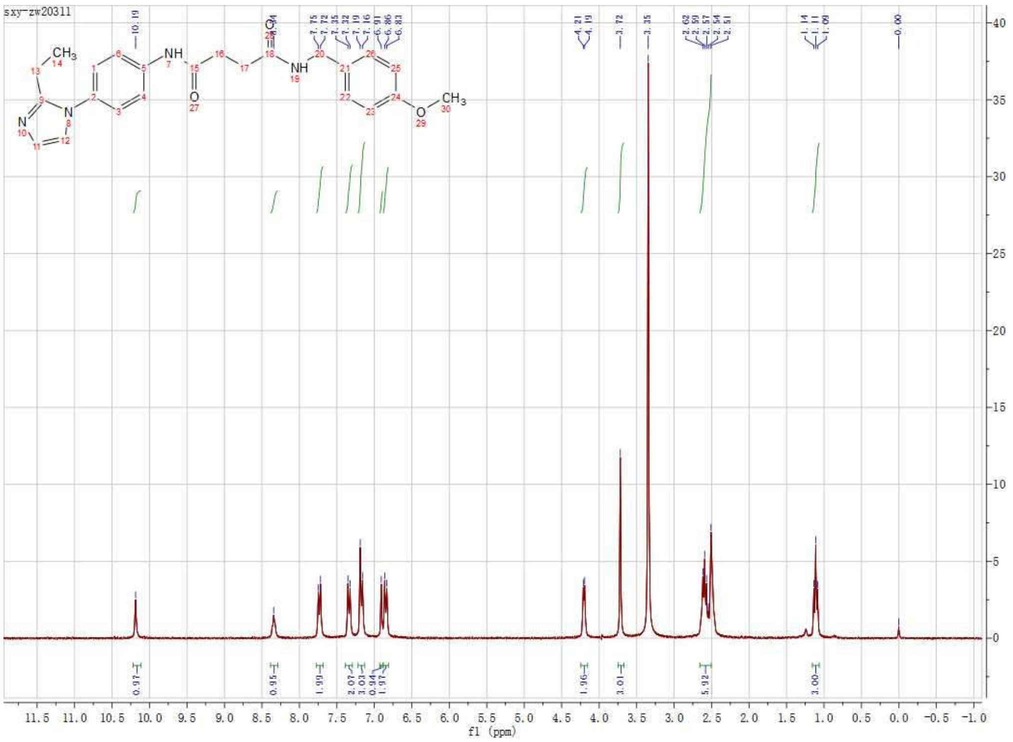

^{13}C-NMR spectrum of Compound **4p**

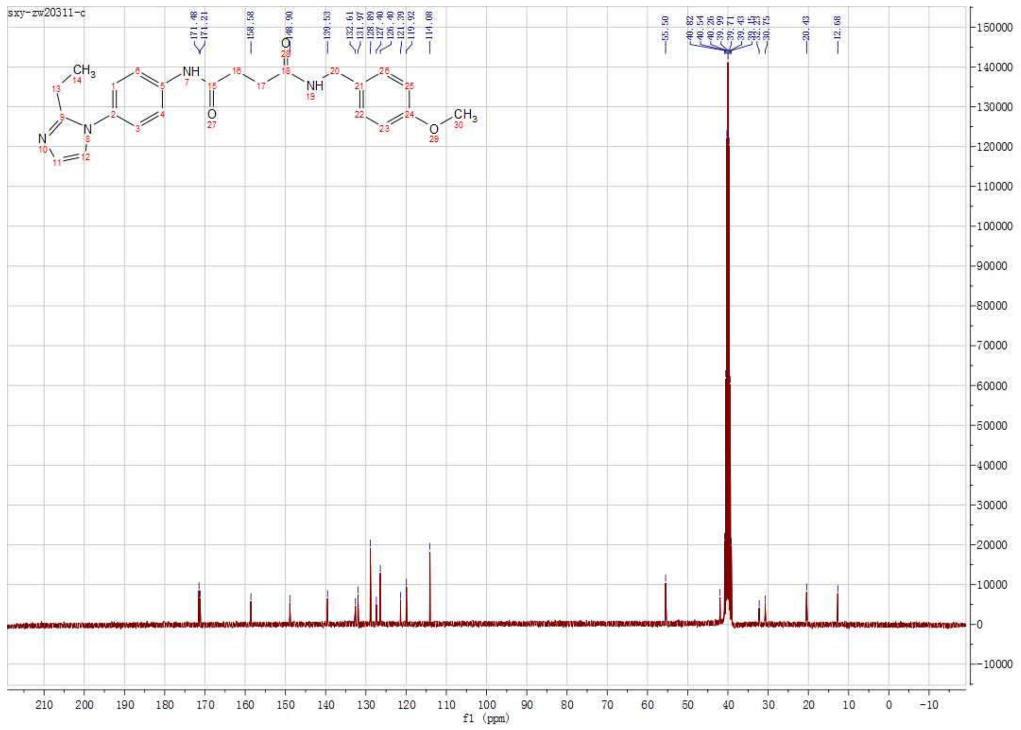

HIGH RESLUTION MASS SPECTROMETRY of Compound **4p**

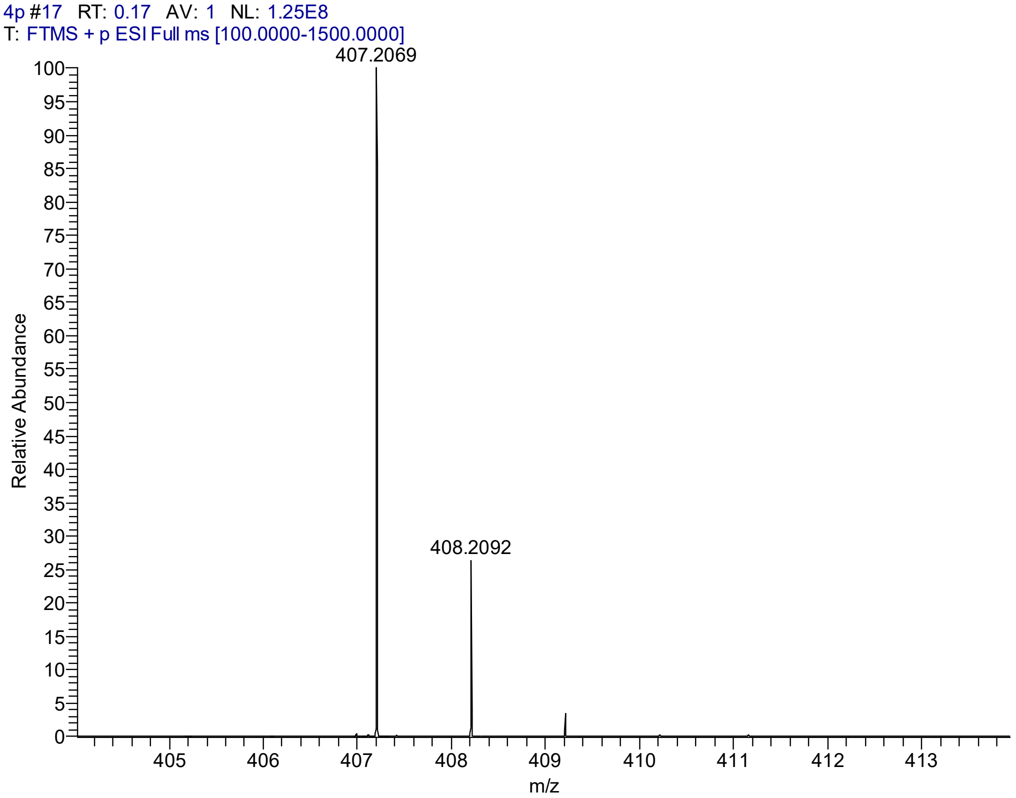

^{1}H-NMR spectrum of Compound **4q**

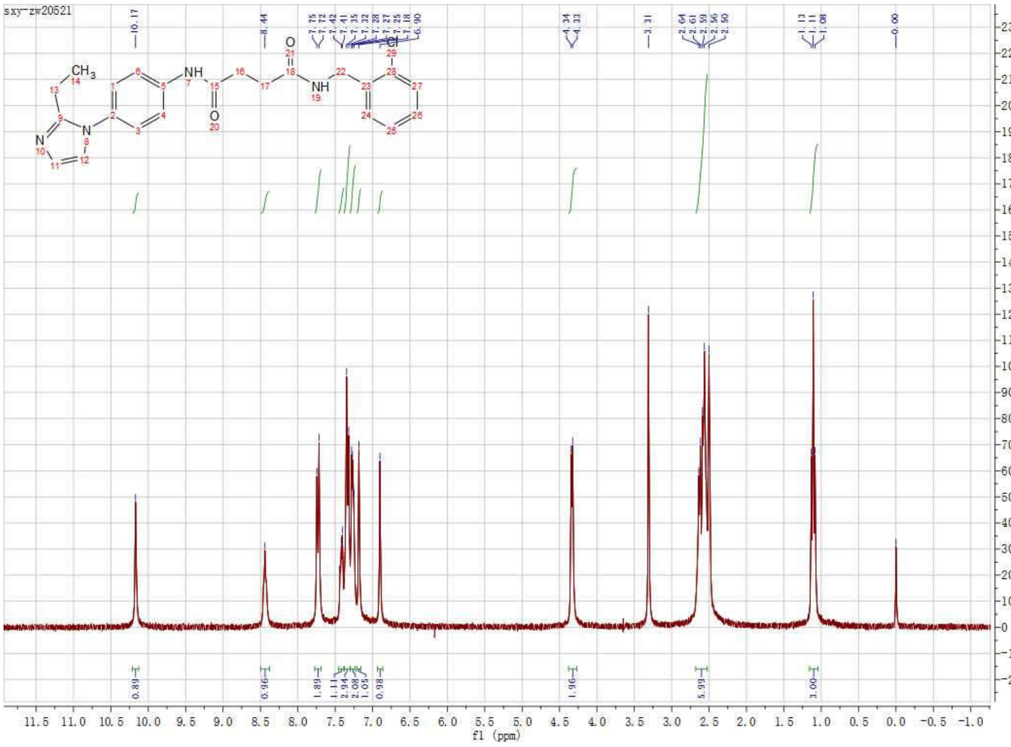

^{13}C-NMR spectrum of Compound **4q**

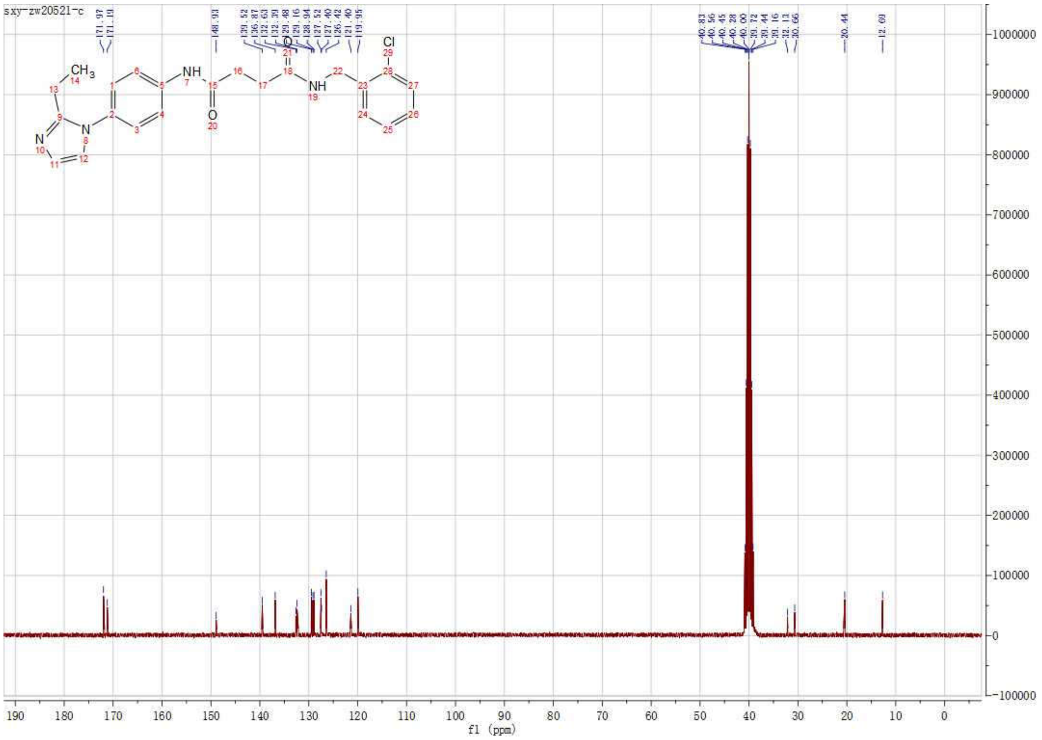

HIGH RESLUTION MASS SPECTROMETRY of Compound **4q**

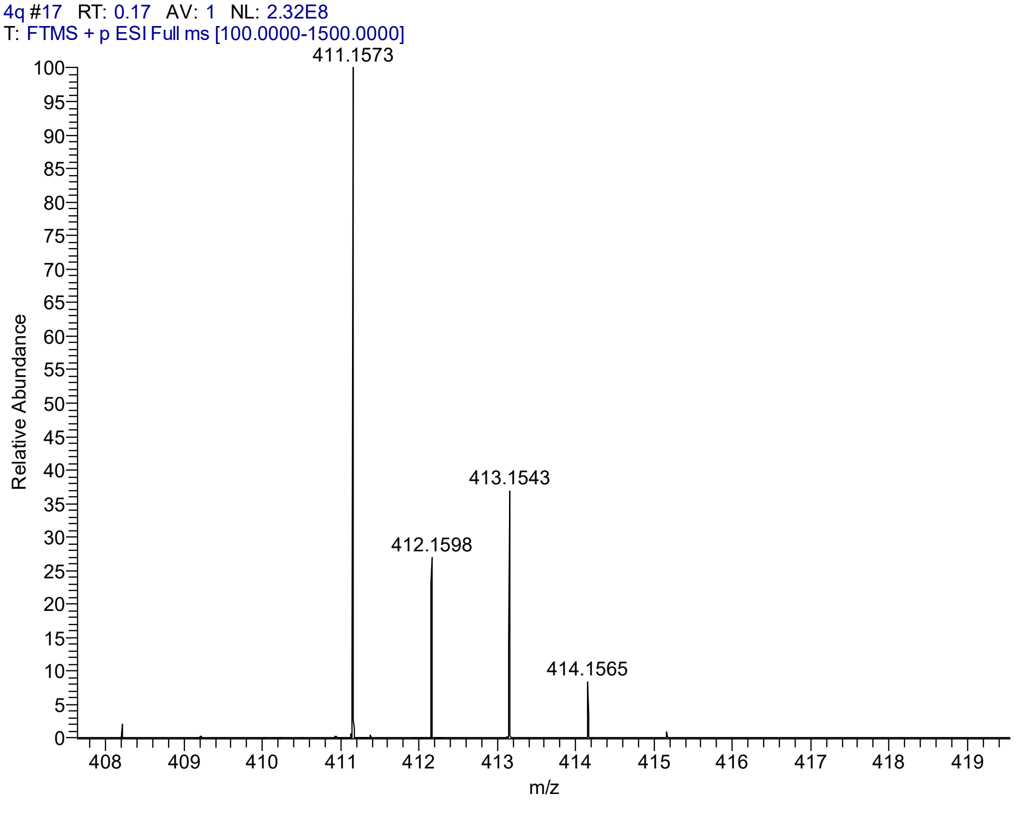

^{1}H-NMR spectrum of Compound **4r**

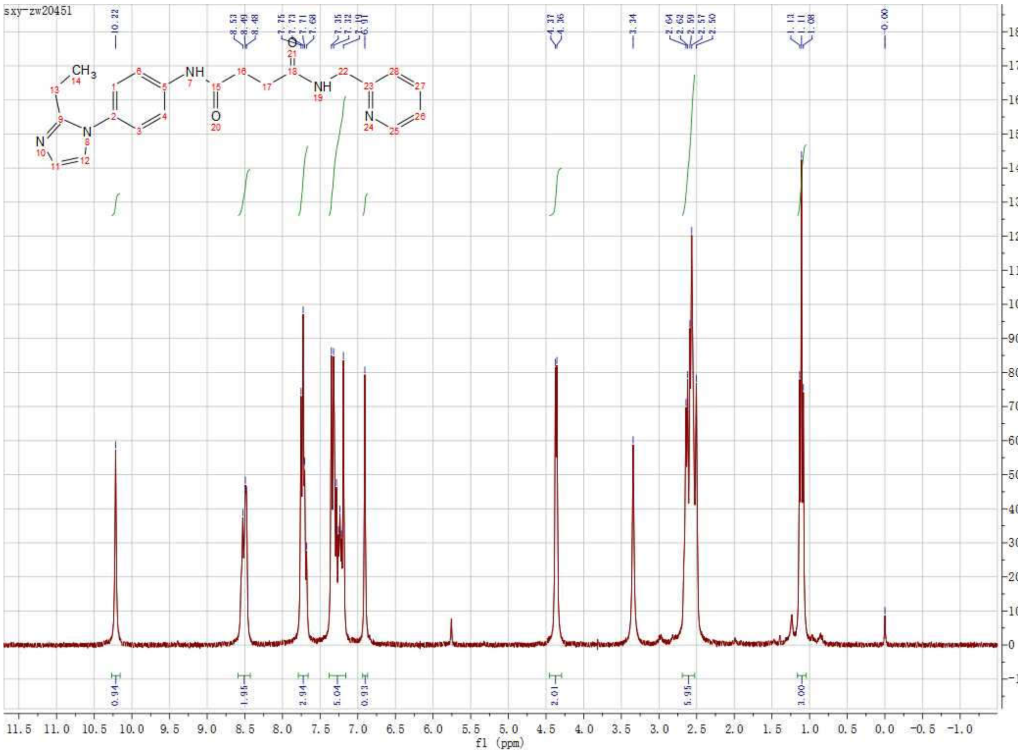

^{13}C-NMR spectrum of Compound **4r**

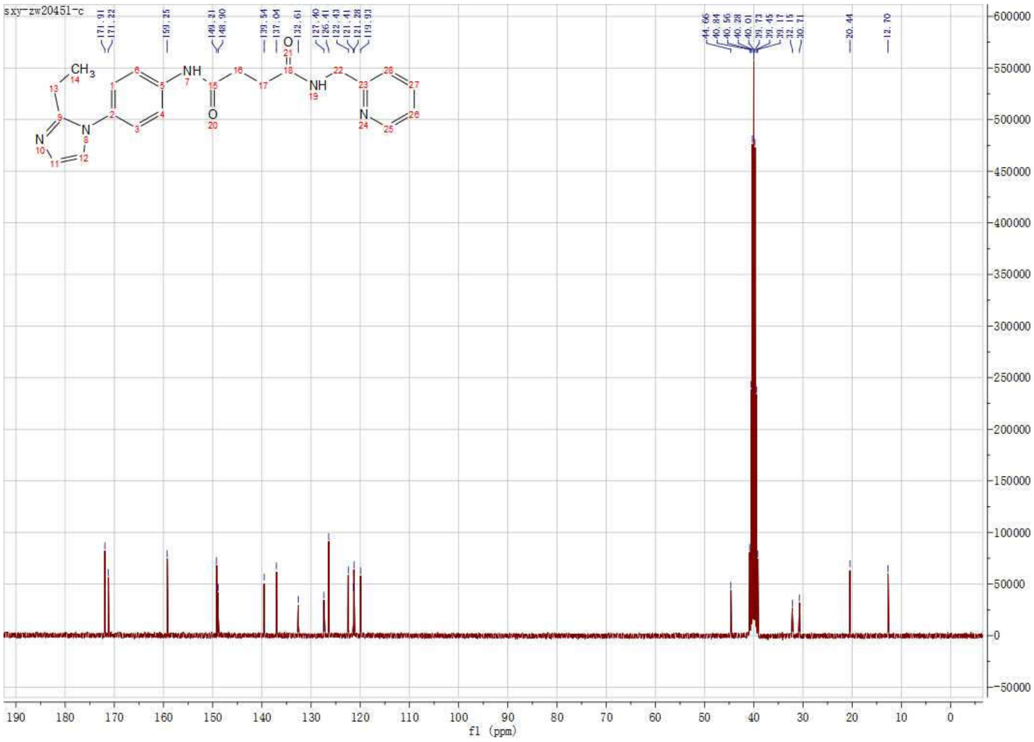

HIGH RESLUTION MASS SPECTROMETRY of Compound **4r**

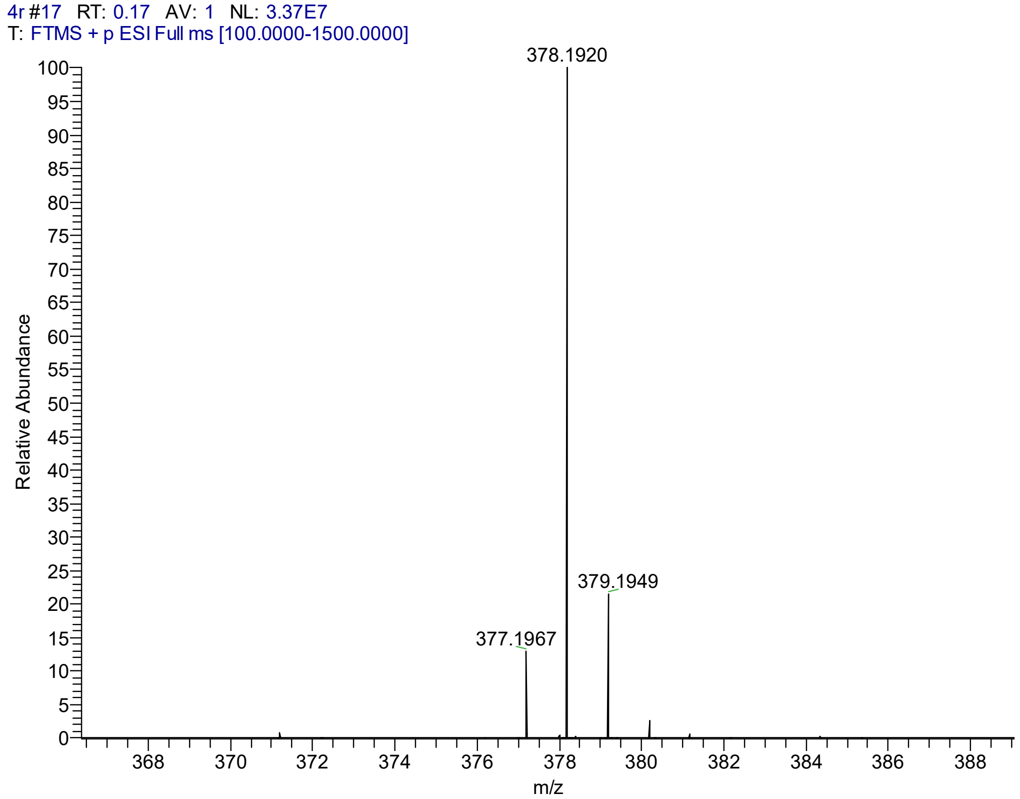

^{1}H-NMR spectrum of Compound **4s**

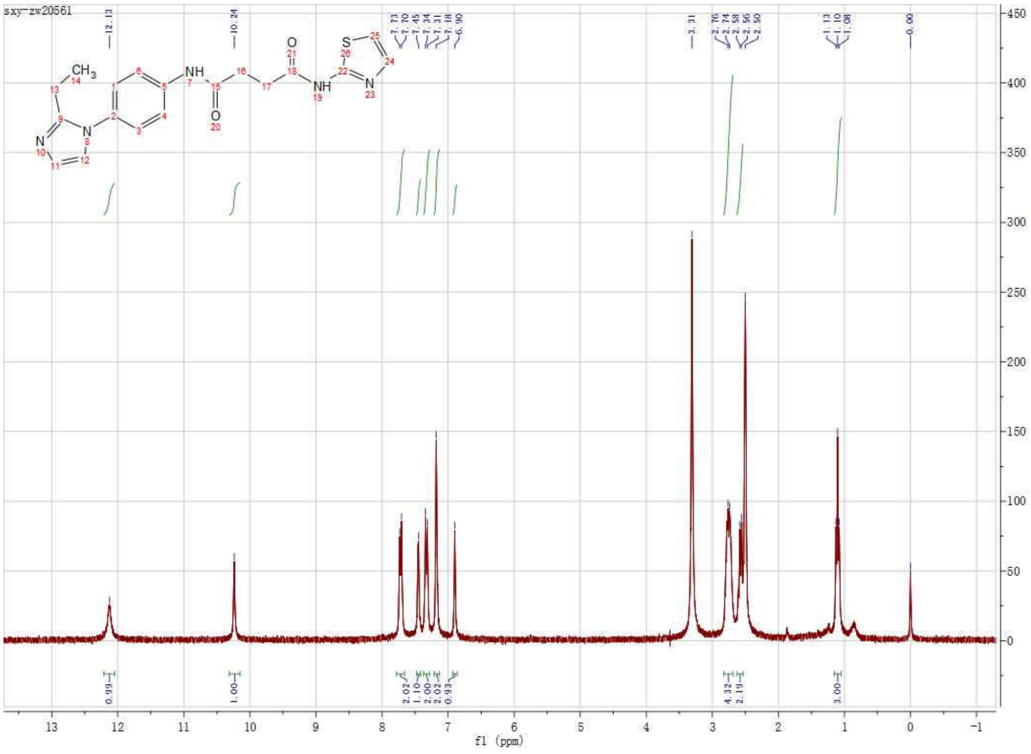

^{13}C-NMR spectrum of Compound **4s**

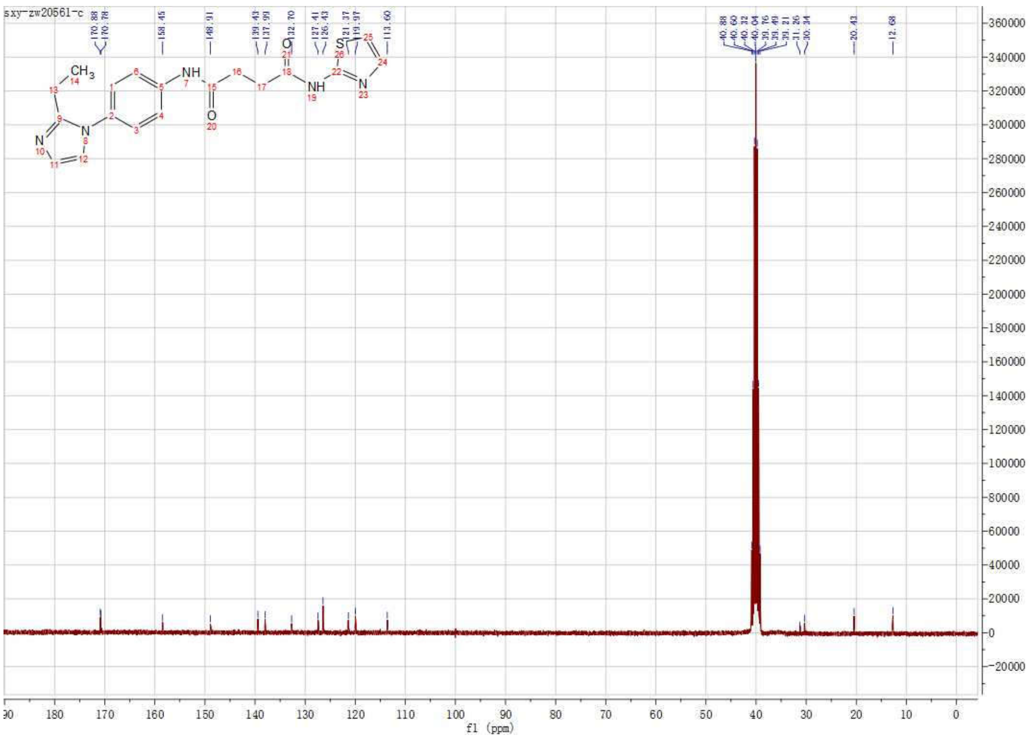

HIGH RESLUTION MASS SPECTROMETRY of Compound **4s**

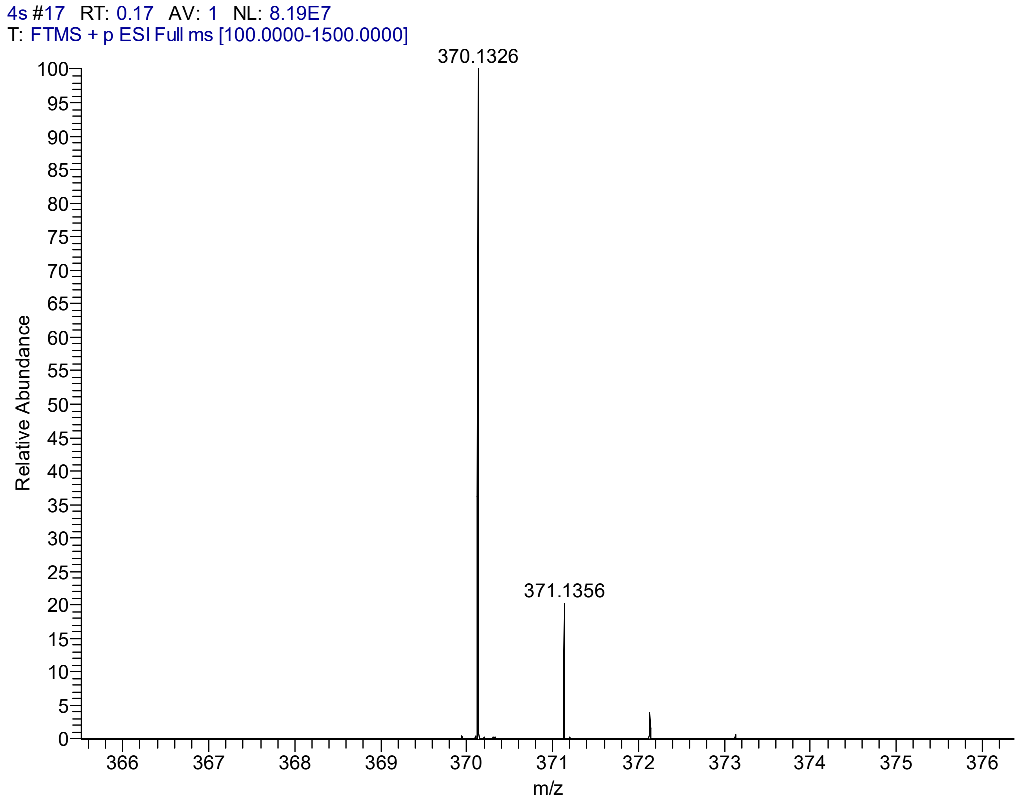

Supplement: Supplementary file 1 [file molecules-25-04293-s001.pdf]
